# The CAMP study: feasibility and clinical correlates of standardized assessments of substance use in a youth psychiatric inpatient sample

**DOI:** 10.1186/s13034-021-00403-4

**Published:** 2021-09-13

**Authors:** Jillian Halladay, Laurie Horricks, Michael Amlung, James MacKillop, Catharine Munn, Zil Nasir, Rachel Woock, Katholiki Georgiades

**Affiliations:** 1 Department of Health Research Methods, Evidence, and Impact, McMaster University, 1280 Main Street West, ON L8S 4S4 Hamilton, Canada; 2 Child and Youth Mental Health Program, McMaster Children’s Hospital, 1200 Main St W, ON L8N 3Z5 Hamilton, Canada; 3 Department of Applied Behavioral Science, Cofrin Logan Center for Addiction Research and Treatment, Addictions Lab at The University of Kansas, 1000 Sunnyside Avenue, KS 66045 Lawrence, United States; 4 Department of Psychiatry and Behavioural Neurosciences, McMaster University, Hamilton, Canada; 5 Peter Boris Centre for Addictions Research, McMaster University/St. Joseph’s Healthcare Hamilton, 100 West 5th St, ON L8N 3K7 Hamilton, Canada; 6 Michael G. DeGroote Centre for Medicinal Cannabis Research, McMaster University, Hamilton, Canada; 7 Resident Affairs, Postgraduate Medical Education (PGME), McMaster University, 1280 Main Street West, ON L8S 4S4 Hamilton, Canada; 8 Hamilton Health Sciences, 1200 Main St W, ON L8N 3Z5 Hamilton, Canada; 9 Offord Centre for Child Studies, McMaster University, McMaster Innovation Park, Suite 201A, 1280 Main Street West, ON L8S 4K1 Hamilton, Canada

**Keywords:** Adolescent, Cannabis, Alcohol drinking, Substance-related disorder, Psychiatric Hospitals

## Abstract

**Background:**

To determine: (a) the feasibility and acceptability of administering a standardized electronic assessment of substance use and other mental health concerns to youth admitted to an inpatient psychiatric unit, and (b) the prevalence and clinical correlates of substance use in this sample.

**Methods:**

The sample included 100 youth between the ages of 13 to 17 years admitted to an inpatient psychiatric unit in Ontario, Canada between September and November 2019 (78% response rate). Youth data were comprised of electronic self-reported assessments (during hospitalization and 6-months following) and chart reviews (99% consented; historical and prospective). Frontline staff completed a self-report survey assessing their perceptions of the need for standardized substance use assessments, training, and interventions on the unit (n = 38 Registered Nurses and Child and Youth Workers; 86% response rate). Analyses included descriptive statistics, correlations, regression, and qualitative content analysis.

**Results:**

Feasibility of standardized youth self-reported mental health and substance use assessments was evident by high response rates, little missing data, and variability in responses. 79% of youth had used at least one substance in their lifetime; 69% reported use in the last 3 months. Substance use was positively correlated with severity of psychiatric symptoms (τb 0.17 to 0.45) and number of psychiatric diagnoses (τb 0.17 to 0.54) at index. Based on prospective and retrospective data, substance use was also positively related to mental health symptom severity at follow-up and repeat mental health related hospital visits. Frontline staff reported a need for standardized assessment, training, and interventions on the unit, indicative of acceptability.

**Conclusions:**

This study demonstrated the feasibility, acceptability and clinical importance of administering a standardized mental health and substance use assessment among youth experiencing psychiatric hospitalization.

**Supplementary Information:**

The online version contains supplementary material available at 10.1186/s13034-021-00403-4.

## Background

Most mental illnesses emerge in childhood and adolescence, and suicide is the second leading cause of death during adolescence [1, 2]. Although substance use disorders (SUDs) often emerge later than other mental illnesses, most individuals who use substances initiate use prior to age 20 [3]. Cannabis and alcohol are two of the most commonly used substances [4], and accumulating evidence suggests use of cannabis and alcohol may precede the onset or worsening of psychiatric and suicide-related outcomes [5–7]. Regardless of temporal sequencing, co-occurrence of mental health and substance use problems is common [8]. Although help-seeking among adolescents with substance use concerns is low, many engage with psychiatric services prior to substance use treatment [9, 10]. This presents a critical opportunity for prevention and early identification in psychiatric settings.

Assessing and addressing substance use may be particularly important during psychiatric hospitalizations given the acuity of youth presentations, access to multidisciplinary teams, and treatment recommendations and community referrals often facilitated upon discharge. However, standardized instruments, designed to assess substance use and mental health concerns, are not routinely administered in youth psychiatric settings [11–14]. There is emerging but limited evidence suggesting that individuals with mental illnesses who use cannabis or alcohol may experience more severe and complex symptoms, greater functional impairment, and poorer prognosis [7, 9, 15, 16]. This evidence is primarily drawn from work in outpatient settings and is not routinely collected as a means to provide robust insight.

When considering youth populations from the perspective of those presenting to health services with substance use concerns, there is data available on the co-occurrence of mental health problems. In Canada between 2017 and 2018, about 70% of youth hospitalizations for substance use involved concurrent psychiatric concerns [17]. Similarly, a majority of adolescents attending a large outpatient substance use program in Toronto, Ontario, endorsed high levels of internalizing (72%) and externalizing (83%) psychopathology [18]. These findings have been replicated among youth attending substance use treatment in the US [10, 19]. Of note, cannabis typically accounts for the largest proportion of substance use related service use among youth [17–19].

There are significant challenges navigating and securing services for youth that address both mental health and substance use in North America [9, 20]. Longstanding gaps in youth addiction services have been recognized by governments and there have been calls for increased capacity to treat SUDs and psychiatric disorders concurrently across all sectors of youth care [20]. Both Canadian and US governments have recognized the need to identify substance use problems early, especially among those with psychiatric concerns, and have indicated a need for integrated and coordinated treatments [21–23]. This is echoed in various clinical best practice guidelines (BPGs) which recommend assessing for substance use prior to diagnosing mental illnesses and treating concurrently if co-presenting [24–27]. Despite the recognition of this problem, this gap in service persists. Common healthcare provider reported barriers to addressing substance use include time constraints, lack of training, stigma, and uncertainty about how to interpret and apply results of screening assessments [13, 28, 29]. Notably, a recent systematic review of concurrent disorder recommendations within existing BPGs found a lack of specificity and consistency regarding recommendations, as well as low levels of rigor and stakeholder input when developing the guidelines [30]. Further, no specific guidelines address the management of youth substance use on inpatient psychiatric units, which were not built or funded to address both issues. As such, further research and stakeholder input are critical to inform guidelines and advocate for funding and system changes where it is most needed.

The Cannabis, Alcohol, Mental Health, and Patterns of Service Use (CAMP) study was a pilot study to determine the feasibility and acceptability of collecting and integrating substance use, mental health, and hospitalization data among youth admitted to an inpatient psychiatric unit through both primary data collection methods (i.e., self-reported youth electronic clinical assessments, stakeholder surveys) and secondary linkages to medical records by research personnel. Our results can inform subsequent: (1) clinical research studies, designed to assess the feasibility, acceptability, utility and cost-effectiveness of integrating routine substance use and mental health assessments directly within clinical practice; and (2) methods for larger scale research studies within clinical programs. The specific feasibility objectives included [31]: (1) *process* outcomes, i.e., ability to recruit (patient willingness); (2) *resource and management* outcomes, i.e., youth and staff burden and extent of missing data, refusal, and retention; (3) *scientific* outcomes, including prevalence and variability in substance use and preliminary insight into correlates between substance use and psychiatric severity (i.e., intensity of symptoms), complexity (i.e., comorbidity), and health service use (i.e., length of stay and readmission); and (4) *staff acceptability* outcomes, including staff perceptions regarding substance use assessment and intervention on the inpatient unit, including its importance, facilitators, and barriers.

## Methods

### Design and setting

The CAMP study was a feasibility observational cohort study conducted on a large Child and Youth Mental Health Inpatient Unit in a large urban city in Ontario, Canada. The unit services youth up to the age of 18 years. The purpose of admission includes emergent psychiatric assessments, crisis stabilization, acute treatment delivery including pharmacological and nonpharmacological approaches (e.g., daily structured individual and/or group psychotherapeutic programming), and coordinated post-discharge planning with community partners. In general, roughly 50% of beds on this unit are occupied by youth experiencing internalizing symptoms (e.g., depression, anxiety, trauma), 23% by youth with primary personality disorder related symptoms (e.g., borderline personality disorder, oppositional defiant disorder), and 27% by highly acute youth (e.g., psychotic and manic episodes). The average length of stay is 7–10 days, appreciating the vast majority of treatment provision occurs post-crisis in the community. Developed 12 years ago, the units focus has been on the acute stabilization of psychiatric presentations. Over the past 12 years, the unit has admitted youth with concurrent disorders, and openly acknowledges it is not a designated concurrent disorders unit and therefore does not provide specific treatments for SUDs.

The study consisted of 4 parts: (1) a self-reported electronic youth assessment during hospitalization; (2) a 6-month follow-up assessment; (3) retrospective (3 years) and prospective (6 months) chart reviews; and (4) frontline staff surveys. The staff component combined cross-sectional and qualitative description designs in survey format [29]. All study objectives and procedures were iteratively refined with feedback from frontline staff, unit leadership, and the Child and Youth Mental Health Research Advisory Committee. Of note, the selected clinical indicators related to severity, complexity, and health service utilization align with provincially defined clinical indicators for child and youth mental health services [32, 33].

### Participants

The target population for the youth component was all youth 12–17 years of age admitted to the unit. The sample included 100 youth recruited on a rolling basis. Youth were excluded if they were: unable to provide informed consent, unable to complete a 30-min assessment (due to attention, cognitive, or safety concerns), or experiencing acute psychotic symptoms based on clinical staff evaluations. Substance use was not required. Recruitment occurred between September 9, 2019 and November 26, 2019. The target population for the staff component was all frontline full-time and part-time Registered Nurses (RNs) and Child and Youth Workers (CYWs) as of September 2020.

### Measures

#### Youth self-report measures

The youth assessment was based on a clinical screening tool used on the adult Concurrent Disorders units in Hamilton (St. Joseph’s Healthcare Hamilton), adapted for youth. To facilitate comparisons, all measures were selected based on pre-piloted and/or psychometrically validated measures for youth used in large population surveys including the Ontario Student Drug Use and Health Survey (OSDUHS) [4] and the Ontario Child Health Study (OCHS) [34]. The assessment measured demographic characteristics, substance use with a particular focus on cannabis and alcohol use, psychiatric symptomatology, and mental health service utilization. The adapted interview tool was piloted and revised to ensure clarity and minimal burden. See Table 1 for a summary of measures (see Additional file 1 for a PDF of the assessment).

**Table 1:** Summary of key measures in the youth electronic assessment

General construct	Specific variables
Demographics	Age, gender, sex, race/ethnicity, immigrant status, subjective social status [4, 48]
Substance use variables	
Cannabis use	• Frequency of use [4, 58] • Symptoms of cannabis use disorder (CUDIT-R) [59] • Age of onset [4, 58] • Percentage of typical THC/CBD, grams per use day, cost per week/month • Coping motives scores [60] • Proportion of time spent using with others [61] • Co-use with alcohol and co-use with tobacco [4]
Alcohol use	• Frequency of any use [4, 58] • Frequency of heavy episodic drinking (HED; 5+ drinks in a sitting) [4] • Symptoms of alcohol use disorder (AUDIT) [62] • Coping motives scores [63] • Proportion of time spent using with others [61]
Smoking	• Frequency of smoking cigarettes/cigars [4, 58] • Frequency of e-cigarette use and types of substances in e-cigarettes [4, 58]
Prescription drug misuse	Frequency of [4, 58, 64]:
	• Prescription stimulants
	• Prescription opioids
	• Prescription sedatives
Other drug use	Frequency of [4, 58, 64]:
	• Cocaine
	• Methamphetamine
	• Solvents
	• Hallucinogens
	• Street opioids
	• Steroids
Psychiatric clinical severity and complexity	
Severity of psychological distress	The Kessler 6 (K6) [65] provided a dimensional measure of non-specific psychological distress. Previously derived cut-offs of ≥ 13 which indicate serious mental illness were used
Internalizing symptom severity	The OCHS Emotional Behavioral Scales (OCHS-EBS) [66] dimensional measure captured symptoms of internalizing disorders including:
	• Major depressive episode (MDE; of note, suicide item removed)
	• Generalized anxiety disorder (GAD)
	• Social phobia (SP)
Externalizing symptom severity	The OCHS-EBS [66] dimensional measure captured symptoms of externalizing disorders including:
	• Oppositional defiant disorder (ODD)
	• Conduct disorder (CD)
	• Attention deficit hyperactivity disorder (ADHD)
Youth derived clinical complexity	Clinical cut-offs for OCHS-EBS disorder scores based on prevalence estimates derived from a diagnostic structured interview in the original OCHS general population sample were used to generate categorical prevalence of disorders [34]. The number of cut-offs youth exceeded were summed to derive number of internalizing, externalizing, and total disorders as indicators of youth reported clinical complexity.
Symptoms of psychosis	A pre-existing symptom scale adapted from the Diagnostic Interview Schedule [67] provided a dimensional measure of symptoms of psychosis

#### Youth chart reviews

Person-level, *health service utilization* data was collected on prior (past 3 years) and follow-up (6 months post-discharge) psychiatric and substance use related emergency department (ED) presentations and inpatient psychiatric admissions at the hospital. The hospital for data collection is the only pediatric hospital in the city but it is possible for youth in surrounding cities to present to EDs at other hospitals and then get transferred to this inpatient unit (i.e., direct admission). ED visits were identified in Canadian Institute for Health Information (CIHI) National Ambulatory Care Reporting System (NACRS) database using the Canadian Emergency Department Information System (CEDIS) presenting complaints alongside the most responsible diagnosis code for each ED encounter at the hospital site. Inpatient admissions and associated length of stay were identified in CIHI Discharge Abstract Database (DAD) by the most responsible discharge diagnosis code for each inpatient encounter. Substance use and mental health codes were included. Data on *severity and complexity* included documentation by clinicians on harm to self, harm to others, property damage, symptoms of psychosis, substance use, and discharge diagnoses (for a complete list of codes and extraction content, see Additional file 2). Substance use information came from existing semi-structured interviews documented in patient charts by either the nurse upon admission to the unit, or the psychiatrist during the psychiatric assessment. Interviews included open-ended questions related to substances used and patterns of use prior to the inpatient admission. However, these interviews were not standardized and did not utilize validated measures, consistent historical timelines, or systemized probes or response options. Additionally, the documentation system had sections with limited character counts.

#### Staff self-report measures

The staff survey was informed by previous research [13, 28, 29] alongside consultations with the CAMP study team, unit management and leadership, and senior frontline staff. The staff survey included 14 closed and open-ended questions related to standardized youth substance use assessment, treatment planning, training/education, and potential barriers and facilitators to addressing these on the unit (see Additional file 3 for a PDF of the survey).

### Recruitment and data collection

#### Youth

The study Research Assistants (RAs) were trained on general reasons for admission, unit staffing model, common clinical presentations, and specific items about their role in the maintenance of environmental safety. Unit staff were informed about the study through emails and staff meetings beginning one month prior to recruitment through to completion of baseline data collection. Patients were recruited primarily through a one-on-one discussion with an RA. Alternative methods included RAs providing a brief study overview during morning group on a semi-weekly basis and study posters. The RAs consulted with nursing staff about eligible patients in advance of meeting with the patient, to ensure eligibility, safety, appropriate timing, and capacity to consent. Data was collected on an iPad using Qualtrics CoreXM, which is a secure online survey platform and database (Qualtrics, Provo, UT). The RA supervised the youth as they completed the assessments. For youth who consented, a 6-month follow-up assessment was sent to their phones and/or emails (with one reminder) and chart reviews were done to obtain information before, during, and after their index hospital admission. Youth were able to consent to partake in 1, 2 or all 3 parts of the study (i.e., baseline, follow-up, chart reviews), and received a $10 gift card for each component (up to $30).

#### Frontline staff

Staff were recruited through personalized cards in their staff mailbox, emails, posters, and reminders during morning rounds. Data was also collected using Qualtrics CoreXM. All staff received a $20 gift card regardless of participation to keep responses anonymous.

### Ethics and reporting guidelines

Ethics approval was obtained from the Hamilton Integrated Research Ethics Board (ID 7075) and study procedures were approved by the Child and Youth Mental Health Research Advisory Committee. Consent to participate was obtained directly from youth, and not parents, in order to mitigate bias in reporting substance use [35, 36], and to maintain parameters of confidentiality. Our focus on capacity rather than age, is consistent with the Tri-Council Policy Statement, Health Care Consent Act, and previous research demonstrating that youth 12 years of age or older are often capable of consent [37]. Methods and reporting follow pilot study guidelines [31], Strengthening the Reporting of Observational Studies in Epidemiology (STROBE) guidelines, and Reporting of Studies Conducted Using Observational Routinely-Collected Health Data (RECORD) guidelines (for reporting checklists, see Additional file 4).

### Statistical analyses

#### Youth component

This paper examines feasibility outcomes [31], predominantly operationalized as: (1) recruitment of 100 youth within 4 months with a response rate greater than 75%; (2) at least 80% of youth consenting to chart reviews and follow-up assessments; (3) over 80% of consenting youth completing their 6-month follow-up assessment; and (4) at least 20% of youth reporting monthly cannabis and/or heavy alcohol use. Thresholds for adequate response rates come from Risk of Bias tools [38]. Using representative general population data [39], we estimated the prevalence of monthly cannabis use to be 1.7 times greater and heavy drinking to be 1.5 times greater for youth experiencing high levels of psychiatric symptomatology, compared to those with no or few symptoms.

Descriptive statistics were used for feasibility outcomes and to characterize the sample including substance use prevalence estimates with 95% Confidence Intervals (CIs) calculated for proportions [31]. Bivariate Kendall’s Tau (τb) correlations using a *p* < 0.05 to denote significance were used to examine associations between self-reported substance use variables and clinical severity and complexity. Logistic regressions were conducted to explore associations between substance use and any ED visit or inpatient admission, adjusted for type of index admission (e.g., whether patients were directly admitted or went through the local ED). Linear regressions were done to examine associations between self-reported substance use at index and psychiatric symptomatology at follow-up, adjusted for symptomatology at index. All analyses were done using complete cases, after pro-rating summative scales for up to 3 missing items.

#### Staff component

Descriptive statistics were used to provide frequencies and averages of closed-ended response options. Two researchers (JH and RW) used qualitative content analysis to code all open-ended data manually through adding index labels, which were then counted and inductively categorized based on regularities and patterns in the topic codes [40]. Final categories evolved through refinement of codes by re-reading, discussions, and consultations with the larger research team [41]. Results from the quantitative and qualitative items were deliberately integrated and merged during the analysis and interpretation phase to obtain a more complete picture of staff perspectives [42].

## Results

### Youth component

#### Response rates and retention

**Fig. 1:**
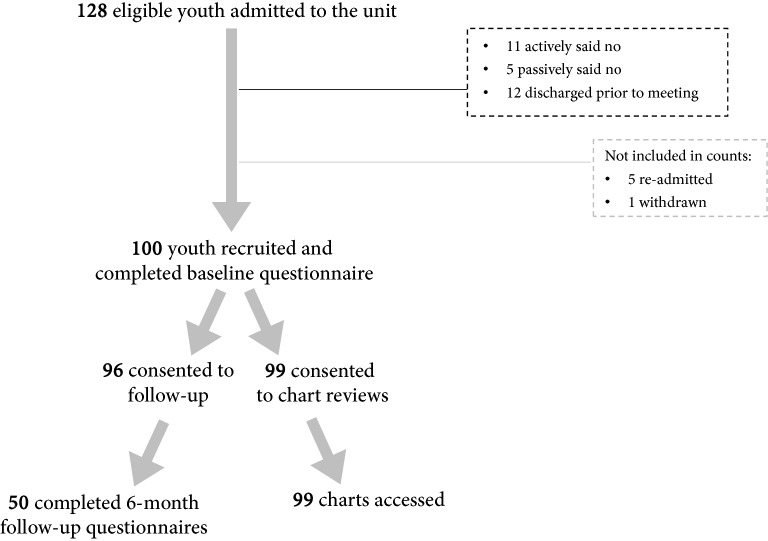
Youth participant flow chart

During the 3-month data collection period, of the 128 youth that met inclusion criteria, 111 were invited to participate in the study, and 100 youth consented to be involved in the study (78% [95% CI 70% to 86%] response rate of all eligible youth, 90% response rate of those invited). For baseline assessments, 77% of youth completed all items with the remaining missing 3 or fewer items. Almost all youth consented to follow-up assessments (96% [CI 92% to 100%]) and chart reviews (99% [CI 97% to 100%]). 50 (52% [CI 42% to 62%]) youth responded to the follow-up assessments within 3 weeks of their 6-month follow-up date.^1^ At follow-up, 78% had complete data with the remaining missing 4 or fewer items. The study surpassed all a priori feasibility criteria, with the exception of the follow-up rate (52% vs. proposed > 80%) which was likely influenced by the COVID19 pandemic. Of note, only higher psychological distress (Odds Ratio [OR] = 0.913, p = 0.034) and prior mental health ED visits (OR = 0.420, p = 0.036) were associated with a lower odds of missing at follow-up; no other indicators of severity, complexity, service use, substance use, or demographic characteristics predicted missingness. See Fig. 1 for a participant flow chart.

#### Recruitment and data collection strategy

Recruitment and data collection processes were efficient and acceptable. Interactions between RAs and staff took on average 5 min per interaction and staff did not express concerns about time taken away from clinical care. RAs typically took 30–40 min to discuss the study and thoroughly review the consent forms with youth. Baseline assessments took youth on average 13 min to complete, with a minimum of 5 and maximum of 33 min (variability due to skip patterns). Of youth included in follow-up assessments, 23 (47%) completed via email and 26 (53%) via smartphones, thus supporting the inclusion of both options.

#### Demographics

Youth were on average 15.4 years of age (age range, 13–17 years). Most youth were female gender (65%). With 2 outliers removed, the average length of stay for index admissions was 8.4 days (shortest 1 day, longest 21 days). In the 3 years prior to index, 44% of the sample had an ED visit for mental health concerns and 40% had a psychiatric admission at the data collection site. In the 6 months following index, 27% re-presented to ED and 24% were re-admitted for mental health concerns at the data collection site. See Table 2 for demographic characteristics.

**Table 2:** Demographic and clinical characteristics of youth participants

Sample characteristics	Mean (SD) or %
Demographic characteristics	
Age	15.4 (1.2)
Perceived social status	5.5 (1.7)
Female sex	82%
Female gender	64%
Transgender and gender diverse	19%
White race/ethnicity	72%
Mixed race/ethnicity	17%
Lived in Canada whole life	91%
One or more parents born outside of Canada	25%
Positive screening on self-reported psychiatric symptom scales	
Generalized anxiety disorder (GAD)	69%
Social phobia (SP)	22%
Major depressive episode (MDE)	50%
Attention deficit hyperactivity disorder (ADHD)	32%
Oppositional defiant disorder (ODD)	37%
Conduct disorder (CD)	32%
At least one internalizing disorder (GAD, SP, and/or MDE)	75%
At least one externalizing disorder (ADHD, ODD, and/or CD)	49%
At least one internalizing and one externalizing disorder	37%
Any internalizing or externalizing disorder	87%
Serious mental illness (K6)	85%
Most responsible physician discharge diagnosis (primary diagnosis, youth only have one)	
Depressive related disorders	29%
Anxiety and obsessive–compulsive related disorders	22%
Trauma and stressor related disorders	21%
Borderline, cluster B, and emotion dysregulation disorders	5%
ADHD and other neurodevelopmental disorders	5%
Other (for complete list, see Additional file 3, available online)	17%
Discharge summary diagnoses^a^	
Depressive related disorders	43%
Anxiety and obsessive–compulsive related disorders	64%
Trauma and stressor related disorders	31%
Borderline personality, cluster B, and emotion dysregulation disorders	41%
ADHD and other neurodevelopmental disorders	20%
Problems with family relations	17%
Eating disorders	12%
SUDs	10%
Other	14%
Number of any discharge diagnoses	2.9 (1.5)
Number of categories of discharge diagnoses (excluding other)	2.5 (1.2)
Hospital contacts prior to and following index	
Any ED visit in prior 3 years	44%
Any ED visit in prior 6 months	25%
Any ED re-presentations in following 6 months	27%
Any admission in prior 3 years	40%
Any admission in prior 6 months	21%
Any re-admission in following 6 months	24%

^a^Discharge summary diagnoses are not mutually exclusive, and youth can have multiple

#### Mental health symptomatology

Using clinician-identified most responsible (one per youth) discharge diagnosis, depressive-related (29%), anxiety and obsessive–compulsive related (22%), and trauma and stressor related (21%) disorders were the most common. When using diagnoses taken from discharge summary notes, in which multiple diagnoses could be identified, the most common disorders were: anxiety and obsessive–compulsive related (64%), depressive related (43%), borderline personality, cluster B, and emotion dysregulation related (41%), and trauma and stressor related (31%). Of note, 10% of youth had a discharge diagnosis of a SUD, none of which were the most responsible diagnosis. Using self-reported symptom scores, 87% surpassed cut-offs for at least one mental health disorder. Specifically, 75% and 49% surpassed thresholds for at least 1 internalizing or externalizing disorder, respectively, with 37% meeting criteria for both. See Table 2 for mental health symptomatology and diagnostic characteristics.

#### Substance use

69% of youth had used at least one substance in the 3 months prior to their psychiatric admission. The most common substances used among youth in the 3 months prior to admission were alcohol, cannabis, tobacco, e-cigarettes, and opioids. Use of multiple substances was common, whereby 50% of youth were using more than one substance prior to admission. Co-use was common, with 24 youth (60%) combining alcohol and cannabis and 25 youth (63%) combining tobacco and cannabis. See Table 3 for prevalence of substance use at index. Self-reported substance use in assessments was higher than documented use in clinical notes—especially when compared to nursing assessments done on admission. These discrepancies could be due to differential reporting by the youth in confidential self-reported assessments versus clinical interviews, but it is more likely that discrepancies arise given differences in content coverage between assessments (i.e., specific questions, timelines, probing, response options, etc.). For example, the electronic self-reported assessment provided examples of each substance type and response options to aid with recall, which were not standardized in clinical interviews.

**Table 3:** Prevalence of youth substance use prior to index admission

Substance	Time period or type of use	Total sample (n = 100)
Alcohol	Lifetime	73% (64 to 82)
	Past 3 months	51% (41 to 61)
	HED past month	29% (20 to 38)
	AUDIT 8–15 “risky”^a^	17 (33% [20 to 46])
	AUDIT ≥ 16 “harmful and high risk”^a^	6 (12% [3 to 21])
Cannabis	Lifetime	66% (57 to 75)
	Past 3 months	50% (40 to 60)
	Daily past month	18% (10 to 26)
	CUDIT 8–11 “hazardous”^a^	5 (10% [2 to 18])
	CUDIT ≥ 12 “possible CUD”^a^	27 (54% [40 to 68])
Tobacco	Lifetime	47% (37 to 57)
	Past 3 months	33% (24 to 42)
	Daily past month	14% (7 to 21)
E-cigarettes	Lifetime	42% (32 to 52)
	Past 3 months	34% (25 to 43)
	Daily past month	14% (7 to 21)
Prescription opioids	Lifetime	22% (14 to 30)
	Past 3 months	18% (10 to 26)
Sedatives	Lifetime	22% (14 to 30)
	Past 3 months	14% (7 t 21)
Prescription stimulants	Lifetime	21% (13 to 29)
	Past 3 months	11% (5 to 17)
Cocaine	Lifetime	18% (10 to 26)
	Past 3 months	8% (3 to 13)
Hallucinogens	Lifetime	22% (14 to 30)
	Past 3 months	14% (7 to 21)
Solvents	Lifetime	10% (4 to 16)
	Past 3 months	4% (0 to 8)
Any prescription drug	Lifetime	35% (26 to 44)
	Past 3 months	24% (16 to 32)
Any illicit substance use	Lifetime	34% (25 to 43)
	Past 3 months	17% (10 to 24)
Any substance use	Lifetime	79% (71 to 87)
	Past 3 months	69% (60 to 78)

Methamphetamines, street opioids, steroids, and synthetic cannabinoids were not included uniquely as prevalence was < 4%

^a^For those who endorsed use in the 3 months prior to index visit

##### Cannabis

The average age of initiation among all youth who reported lifetime cannabis use was 13.3 years. Of the 50% of youth who reported cannabis use in the 3 months prior to admission, 32 (64%) had CUDIT-R scores indicative of hazardous cannabis use (mean = 12.4; SD = 7.4) and 23 (46%) had recently thought about cutting down or stopping use. Of the 45% of youth who endorsed past month use, 25 (55%) reported using alone half of the time or more, 35 (78%) reported using to cope most or all of the time, and 18 (40%) reported daily use. Notably, frequency of cannabis use and using for coping purposes accounted for 60% of the variance in CUDIT-R scores. Prevalence of monthly cannabis use was 3.2 times greater than prevalence in the general population of grade 7–12 students (14.1%) [4], surpassing a priori feasibility thresholds.

##### Alcohol

Of the 51% who reported using alcohol in the 3 months prior to admission, 23 (47%) had AUDIT scores indicative of hazardous alcohol use (mean = 8.4, SD = 6.3). Of these youth, 12 (24%) reported someone being injured as a result of their drinking and 7 (14%) were currently concerned about their drinking. Of the 46% of youth who endorsed past month use, 11 (24%) reported using alone half of the time or more, 30 (65%) reported using to cope most or all of the time, and 29 (63%) reported heavy episodic drinking (HED). Questions regarding past month drinking and HED alongside drinking coping motives explained 58% of the variance in AUDIT scores. Prevalence of monthly HED was 1.9 times greater than the general population (15%) [4], surpassing a priori feasibility thresholds.

##### Cigarettes and E-cigarettes

14% endorsed daily use of tobacco cigarettes and 14% endorsed daily use of e-cigarettes. Types of e-cigarettes were clarified at follow-up, where most youth reported using e-cigarettes with nicotine (79%) and about a third (32%) reported use with cannabis.

##### Other drug and prescription drug misuse

When other drugs were used in the 3 months prior to admission, most youth endorsed using the substance 1 or 2 times with no more than 3% endorsing 10 or more times for any individual substance (3% cocaine, 2% solvents, 2% hallucinogens). Misuse of prescription drugs 10 or more times was more common with youth endorsing frequent use of prescription stimulants (4%), sedatives (5%), and/or opioids (5%).

#### Clinical correlates of substance use

The magnitude, significance, and precision of effects varied across different substance types (e.g., alcohol, cannabis, cigarettes, e-cigarettes, prescription, other) and substance-related variables (e.g., frequency, AUDIT/CUDIT-R, coping motives, using alone) for different clinical indicators. However, significant correlations emerged between at least one substance use variable and: (1) self-reported externalizing (all substances; significant τb from 0.17 to 0.45) and internalizing symptomatology (select substances; significant τb from 0.17 to 0.40); (2) clinician-reported aggressive behaviors (most substances; significant τb from 0.21 to 0.32); (3) number of youth-reported psychiatric disorders (all substances; significant τb from 0.19 to 0.43); (4) number of physician-reported discharge diagnoses (select substances; significant τb from 0.17 to 0.54); (5) mental health related ED visits 3 years prior and 6 months after index (select substances); and (6) psychiatric admissions in the 3 years prior to index (all substances). Additionally, those who completed the 6-month follow-up who used cannabis, alcohol, cigarettes, or e-cigarettes prior to index endorsed significantly higher psychiatric symptoms at follow-up. This serves as preliminary exploratory evidence of correlations between substance use and clinical severity, complexity, service use, and poorer prognosis. See Table 4 for select correlations between substance use and severity and complexity variables. More comprehensive details and results are presented in Additional file 5.

**Table 4:** Selected Kendall’s tau correlations between substance variables and clinical severity and complexity outcomes

	Youth-reported psychiatric symptomatology as per OCHS-EBS							Physician-reported	
	SP	GAD	MDE	ADHD	ODD	CD	Total # surpassing clinical thresholds	Aggressive threats and behaviors	Total # of discharge diagnoses based on categories
Frequency									
Cannabis	− 0.076	0.007	0.075	0.128	0.266^b^	0.309^b^	0.212^b^	0.227^a^	0.117
Alcohol	0.054	0.038	0.105	0.073	0.210^b^	0.250^b^	0.200^a^	0.212^a^	0.093
Cigarette	0.005	0.069	0.071	0.185^a^	0.295^b^	0.447^b^	0.280^b^	0.324^b^	0.167
E-cigarette	− 0.021	0.05	0.107	0.14	0.259^b^	0.300^b^	0.284^b^	0.136	0.141
Prescription	0.208^a^	0.245^b^	0.279^b^	0.168^a^	0.230^b^	0.319^b^	0.360^b^	0.262^b^	0.197^a^
Other	0.081	0.137	0.174^a^	0.206^a^	0.274^b^	0.374^b^	0.341^b^	0.282^b^	0.200^a^
Coping motives									
Cannabis coping motives	0.293^b^	0.398^b^	0.206	0.001	0.052	0.04	0.229^a^	− 0.068	0.358^a^
Alcohol coping motives	0.128	0.302^b^	0.236^a^	0.320^b^	0.299^b^	0.316^b^	0.412^b^	0.159	0.540^b^
Substance use disorder scores									
CUDIT total score	− 0.094	0.015	0.072	− 0.041	0.002	− 0.005	0.005	0.039	0.116
AUDIT total score	0.02	0.123	0.186	0.289^b^	0.336^b^	0.444^b^	0.396^b^	0.281^a^	0.442^b^
Using substances with others									
Using cannabis with others	− 0.172	− 0.14	− 0.112	0.049	0.054	0.108	− 0.043	− 0.085	0.049
Using alcohol with others	−0.103	− 0.167	− 0.067	− 0.111	− 0.143	− 0.117	− 0.187	− 0.113	− 0.205

More detailed results in Additional file 5, available online

^a^Correlation is significant at the 0.05 level (2 tailed)

^b^Correlation is significant at the 0.01 level (2-tailed)

### Staff component

There was an 86% response rate (37/43) with roughly half RNs (49%) and half CYWs (51%). Over half (54%) of the staff participating in the survey had been working on the unit 5 years or longer with only 2 staff reporting less than 1 year experience. The main findings were that: (1) staff believe substance use is important and common among youth on the unit and want to improve how they assess and address substance use; (2) staff have ideas about how to facilitate improvements in quality of care including greater standardization of assessments and interventions, separate cohorting and staffing for youth with more severe co-occurring problems, more direct substance related interventions, and more indirect facilitation of appropriate and supportive conversations; and (3) staff want more education and training to increase knowledge, confidence, and standardization of practices. Of note, lack of training (81%) and time pressures (64%) were the most commonly reported barriers to comprehensive assessment while facilitators included standardization, adding designated spaces in documentation, and training on conducting assessments and addressing positive screens.

## Discussion

The CAMP study examined the feasibility of administering a standardized electronic assessment to measure mental health and substance use on an inpatient youth psychiatric unit and provides insight into the prevalence and correlates of substance use among youth in this acute setting. Collecting this data as part of a research study proved feasible, with high recruitment and response rates, and little participant and staff burden. The high prevalence of substance use provides evidence of the feasibility of general consecutive sampling and reinforces the importance of routine substance use assessments within this context.

Overall, comorbid substance use was the norm, not the exception. A majority of youth had used at least one substance prior to their admission, and substance use correlated with more severe psychiatric symptoms, greater complexity, and more mental health related hospital visits. Youth using substances were often using in ways that have been associated with higher risk of experiencing substance-related problems, including early age of initiation, frequent use, using multiple substances, using alone or for coping purposes, and co-using substances. Despite the unit not being designated as a concurrent disorders unit, youth with substance concerns are admitted. As such, frontline staff recommended adopting a comprehensive approach to substance use among youth admitted to hospital for psychiatric concerns, including adoption of standardized assessments, more training, and enhanced patient conceptualization and intervention which include substance use considerations. Standardized screening and assessments can facilitate efficient identification of patients requiring more thorough SUD clinical assessments or immediate withdrawal management and can support comprehensive patient conceptualization, integrated treatment planning, and referral pathways.

Prevalence and frequency of substance use far surpassed that found in the general population of Ontario youth in grades 7–12. Not only were youth in this study more likely to use substances, but these youth also reported more frequent use, more co-use of substances, and more symptoms related to Alcohol Use Disorder and Cannabis Use Disorder compared to the general population. In particular, almost 1 in 5 youth in the present study reported using cannabis daily and 1 in 7 smoked tobacco products daily, frequencies which are roughly 8 to 9 times greater than general population estimates [4]. Further, this sample reported an age of initiation of cannabis about 2 years younger than the general population (13.3 CAMP vs. 15.4 OSDUHS) and similar to the age of initiation among youth who present to an outpatient concurrent disorders program in Toronto (13.6) [18]. Earlier age of cannabis initiation has been related to a greater likelihood of using multiple substances and developing a SUD [43, 44], experiencing cognitive impairment, lower academic achievement, and dropping out of school [43, 45, 46], having more criminal and legal involvement, and experiencing more concurrent mental health symptomatology [47]. Although there are differences in sampling strategies and characteristics, this provides general evidence of higher prevalence and risky use in clinical samples consistent with existing studies of youth with high levels of psychiatric symptomatology and suicidality [5–8, 48, 49].

Most clinical guidelines indicate the need to assess the role of substances prior to diagnosing and determining treatment for mental illnesses [24–27]. This study demonstrates it is feasible to collect self-reported substance use data electronically from youth experiencing acute psychiatric concerns. Electronic assessments have shown validity, acceptability, and greater efficiency as compared to clinical interviews [50]. Further, the high frequency of substance use seen in this sample demonstrate that a non-negligible proportion of youth admitted to the hospital for psychiatric concerns may be at risk of withdrawal during admission [51]. The most common withdrawal symptoms for cannabis and nicotine are behavioral and emotional, which may bias diagnostic assessments and interfere with care while on an inpatient unit if substance use is not assessed systematically and comprehensively [51]. Thus, screening and assessment should not be reserved only for research studies but rather must be integrated into routine clinical care and treatment planning.

Given neurodevelopmental vulnerability, any and all substance use among adolescents merits clinical intervention, especially among those with comorbid psychiatric concerns [26]. Early intervention has the potential to reduce the severity and persistence of substance use related problems [52]. The inpatient unit also provides a unique opportunity where motivation to change behavior may be higher and access to substances is limited, likely resulting in at least temporary cessation of use. Further, given there is evidence that youth present to mental health services before substance use services [9, 10], psychiatric hospitalizations may present a key opportunity for early intervention and/or referral to treatment. There is a critical need for further research of substance use on youth in psychiatric inpatient settings to inform the development of best practice guidelines and standardized clinical practices.

The existing study was a pilot study of 100 youth and 38 RNs and CYWs at a single institution. Generalizability of findings pertaining to youth is limited due to the small sample size, predominately female sex and White race, and data collection and visit history only obtained at one hospital site. While youth 12 years of age were eligible to participate, no 12 year olds were recruited into the study. This is likely due to the older age distribution of youth admitted to the inpatient units in Canada, where 15–17 year olds account for the highest rates of psychiatric hospitalizations (72%) [17]. Additionally, no youth with a diagnosis of psychosis or bipolar disorder were included in the final sample. In Canada and the US, depression is typically the most common reason for psychiatric hospitalization among adolescents [33, 53–56], which was also found in our sample. In 2019 across Canada, psychotic disorders represented a small proportion of psychiatric admissions for adolescents (< 5%) [17]. Thus, we believe our sample is representative of the majority of adolescent psychiatric hospitalizations in Canada but does not generalize to a small proportion of youth unable to safely and cognitively consent or provide accurate histories, potentially due to young age (≤ 12) and altered mental status such as acute symptoms of mania and/or symptoms of psychosis (based on our study inclusion criteria). Future studies should consider developing and evaluating alternative assessments methods for patients who do not meet these criteria. Further, although we had high frontline staff response rates, generalizability of the staff results is also limited due to the small sample size at a single institution, in addition to only including regular full-time and part-time RNs and CYWs (to preserve anonymity). Future work should include staff feedback from the broader multidisciplinary and leadership team. Overall, recruitment rates were high among those meeting eligibility criteria, increasing confidence in the local representativeness of the sample.

Regarding measurement, gold standard urine drug screens, timeline follow-back, and clinical diagnostic interviews were not used to assess substance use and mental health concerns, but psychometrically validated measures were used alongside chart reviews providing multiple sources for information. Social desirability bias is of particular concern for self-reported data and may have resulted in underestimations of substance use [57]. Although we were unable to completely eliminate risk of social desirability bias, strategies to mitigate bias were used including exclusively requiring youth consent to participate (and not parent) [35, 36], using self-reported as opposed to interview-administered measures, and incorporating reminders about privacy and confidentiality during the consent process and embedded reminders throughout the assessment [12]. Of note, the willingness to complete may have been influenced by confidentiality and the provision of a $10 incentive, which is not viable in routine clinical practice. However, information collected directly by clinical staff can support direct use of this data to inform treatment planning, referrals, and shared decision making with patients that may increase patient engagement without the need for an incentive. Additionally, clinical correlations should be interpreted as preliminary evidence and require further examination in larger samples with multivariable adjustments for potential confounders.

## Conclusions

In conclusion, the present study found that a majority of youth presenting to an inpatient psychiatric unit were engaging in recent substance use, often involving multiple substances, and provides preliminary evidence which supports the use of standardized substance use and mental health assessments during youth psychiatric hospitalizations. Subsequent studies should examine the feasibility and associated costs of having clinicians conduct standardized assessments, versus research assistants. Frontline staff in this study saw the need for standardized comprehensive assessments to improve clinical conceptualization and quality of care. By embedding standardized assessments directly into clinical practice, data becomes useful for: (1) direct patient care, by informing patient conceptualization, treatment pathways, and discharge planning; (2) program evaluation, by characterizing patients and providing insight into quality improvement strategies; and (3) enabling comprehensive and sustainable integration of research. Future work should include co-development and refinement of standardized assessments and related clinical uses with youth, staff, and their families. Combining research and clinical practice will facilitate bridging current policy and clinical gaps while efficiently addressing and mitigating critical research gaps.

## Supplementary Information

**Additional file 1.** Youth baseline survey.

**Additional file 2.** Chart reviews.

**Additional file 3.** Staff survey.

**Additional file 4.** Reporting guidelines.

**Additional file 5.** Detailed methods and results.

## Data Availability

The data used for this study are not publicly available due to its sensitive clinical nature. Data are available from the corresponding author upon reasonable request and will be subject to further ethics approval.

## Abbreviations

ADHD: Attention Deficit Hyperactivity Disorder
AUDIT: Alcohol Use Disorder Identification Test
BPG: Best Practice Guideline
CAMP: Cannabis, Alcohol, Mental health, and Patterns of service use
CD: Conduct Disorder
CEDIS: Canadian Emergency Department Information System
CIHI: Canadian Institute for Health Information
CYW: Child and Youth Worker
CUDIT-R: Cannabis Use Disorder Identification Test-Revised
DAD: Discharge Abstract Database
ED: Emergency Department
GAD: Generalized Anxiety Disorder
HED: Heavy Episodic Drinking
K6: Kessler-6
MDE: Major Depressive Episode
NACRS: National Ambulatory Care Reporting System
OCHS: Ontario Child Health Study
OCHS-EBS: Ontario Child Health Study-Emotional Behavioural Scales
ODD: Oppositional Defiant Disorder
OSDUHS: Ontario Student Drug Use and Health Survey
RA: Research Assistant
RECORD: REporting of studies Conducted using Observational Routinely-collected health Data
RN: Registered Nurse
SP: Social Phobia
STROBE: Strengthening the Reporting of Observational Studies in Epidemiology
SUD: Substance Use Disorder

## Footnotes

1 1 youth submitted 2 follow-up assessments with multiple discrepancies and another youth submitted 2 months after their follow-up time (not included in follow-up rates). Thus, the follow-up analyses were based on a sample size of n = 49.
