# Supplementary material for: The CAMP study: feasibility and clinical correlates of standardized assessments of substance use in a youth psychiatric inpatient sample
Source: Child Adolesc Psychiatry Ment Health. 2021 Sep 13;15:48. doi: 10.1186/s13034-021-00403-4 (PMC8439003; doi:10.1186/s13034-021-00403-4)
Supplement: Supplementary file 1 — Additional file 1. Youth baseline survey. [file 13034_2021_403_MOESM1_ESM.pdf]

The CAMP Study

Survey Flow

Standard Pt ID (1 question)
Introduction *(4 Questions)*<br>About you *(3 Questions)*<br>Cannabis use *(21 Questions*<br>Alcohol use *(17 Questions)*<br>Mental health *(3 Questions)*<br>Tobacco *(3 Questions)*<br>Other drugs *(1 Question)*<br>Service Use (2 Questions)<br>Demographics (Extended) *(4 Questions)*<br>Final statement

---

Start of block: Introduction

Thank you for taking the time to complete this questionnaire as part of The CAMP Study. This questionnaire is going to ask you about your experiences with substance use, your mental health, and whether you have previously gotten help for substance use or mental health concerns.

Often, we will be asking you about your experiences during the 3 months or 4 weeks before your current hospital admission.

Try to think of something that was going on about 3 months before your admission (like a party, holiday, activity, event, birthday, or something else to help you remember this period of time).

- ☐ I have something in mind (1)

Try to think of something that was going on about 4 weeks before your admission (like a party, holiday, activity, event, birthday, or something else to help you remember this period of time).

- ☐ I have something in mind (1)

Keep those time periods in mind as you answer the questions. Answer the questions to the best of your ability - there is no right or wrong answer.

We really appreciate your input!

End of block: Introduction

Start of block: About you

D1. How old are you?
- ☐ 11 (11)
- ☐ 12 (12)
- ☐ 13 (13)
- ☐ 14 (14)
- ☐ 15 (15)
- ☐ 16 (16)
- ☐ 17 (17)
- ☐ 18 (18)

D2. How would you best describe your gender identity?
- ☐ Female (1)
- ☐ Male (2)
- ☐ Non-binary (3)
- ☐ Unsure or questioning (4)
- ☐ You do not have an option that applies to me (5)

D3. Were you born male or female?
- ☐ Female (1)
- ☐ Male (2)

End of block: About you

Start of block: Cannabis use

C1. Have you ever used cannabis (also known as marijuana, "weed", "pot", "grass", "hashish", "hash oil", etc.)?
- ☐ Yes, I used cannabis in the 3 months before my admission (1)
- ☐ Yes, I have used cannabis but not in the 3 months before my admission (2)
- ☐ No, I have never used cannabis (0)

Display This question:
If C1 = Yes, I used cannabis in the 3 months before my admission

CUDIT1. In the 3 months before your admission, how often did you use cannabis?
- ☐ Never (0)
- ☐ Monthly or less (1)
- ☐ 2 to 4 times a month (2)
- ☐ 2 to 3 times a week (3)
- ☐ 4 or more times a week (4)

Display This question:
If C1 = Yes, I used cannabis in the 3 months before my admission
And CUDIT1 != Never

CUDIT2. How many hours were you "stoned" on a typical day when you had been using cannabis?
- ☐ Less than 1 (0)
- ☐ 1 or 2 (1)
- ☐ 3 or 4 (2)
- ☐ 5 or 6 (3)
- ☐ 7 or more (4)

Display This question:
If C1 = Yes, I used cannabis in the 3 months before my admission
And CUDIT1 != Never

CUDIT3. How often during the **3 months before your admission** did you find did you find you were not able to stop using cannabis once you had started?

- ☐ Never (0)
- ☐ Less than monthly (1)
- ☐ Monthly (2)
- ☐ Weekly (2)
- ☐ Daily or almost daily (4)

Display This question:
If C1 = Yes, I used cannabis in the 3 months before my admission
And CUDIT1 != Never

CUDIT4. How often during the **3 months before your admission** did you fail to do what was normally expected from you because of using cannabis?

- ☐ Never (0)
- ☐ Less than monthly (1)
- ☐ Monthly (2)
- ☐ Weekly (3)
- ☐ Daily or almost daily (4)

Display This question:
If C1 = Yes, I used cannabis in the 3 months before my admission
And CUDIT1 != Never

CUDIT5. How often during the **3 months before your admission** did you devote a great deal of your time to getting, using, or recovering from cannabis?

- ☐ Never (0)
- ☐ Less than monthly (1)
- ☐ Monthly (2)
- ☐ Weekly (3)
- ☐ Daily or almost daily (4)

Display This question:
If C1 = Yes, I used cannabis in the 3 months before my admission
And CUDIT1 != Never

CUDIT6. How often during the **3 months before your admission** did you have a problem with your memory or concentration after using cannabis?

- ☐ Never (0)
- ☐ Less than monthly (1)
- ☐ Monthly (2)
- ☐ Weekly (3)
- ☐ Daily or almost daily (4)

Display This question:
If C1 = Yes, I used cannabis in the 3 months before my admission
And CUDIT1 != Never

CUDIT7. How often during the **3 months before your admission** did you use cannabis in situations that could be physically hazardous, such as driving, operating machinery, or caring for children?

- ☐ Never (0)
- ☐ Less than monthly (1)
- ☐ Monthly (2)
- ☐ Weekly (3)
- ☐ Daily or almost daily (4)

Display This question:
If C1 = Yes, I used cannabis in the 3 months before my admission
And CUDIT1 != Never

CUDIT8. Have you ever thought about cutting down, or stopping, your use of cannabis?

- ☐ Never (0)
- ☐ Yes, but not in the 3 months before my admission (2)
- ☐ Yes, during the 3 months before my admission (4)

Display This Question:
If C1 = Yes, I used cannabis in the 3 months before my admission
Or C1 = Yes, I have used cannabis but not in the 3 months before my admission

Cage. How old were you the first time you used cannabis?

- ☐ 9 or younger (9)
- ☐ 10 (10)
- ☐ 11 (11)
- ☐ 12 (12)
- ☐ 13 (13)
- ☐ 14 (14)
- ☐ 15 (15)
- ☐ 16 (16)
- ☐ 17 (16)
- ☐ 18 (18)

Display This Question:
If C1 = Yes, I used cannabis in the 3 months before my admission

CM. in the **4 weeks before your admission**, how often did you use cannabis?

- ☐ Never (0)
- ☐ Once (1)
- ☐ 2 to 3 days (2)
- ☐ 1 to 2 days per week (3)
- ☐ 3 to 4 days per week (4)
- ☐ 5 to 6 days per week (5)
- ☐ Once each day (6)
- ☐ More than once each day (7)

Display This Question:
If CM = Once
Or CM = 2 to 3 days
Or CM = 1 to 2 days per week
Or CM = 3 to 4 days per week
Or CM = 5 to 6 days per week
Or CM = Once each day
Or CM = More than once each day

Cgrams. Please use the images below to help you answer the next question. The image is not to scale and the dollar bill and bottle cap are included to help you visualize the size.

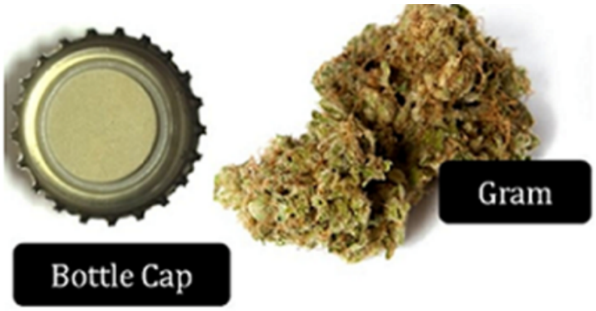

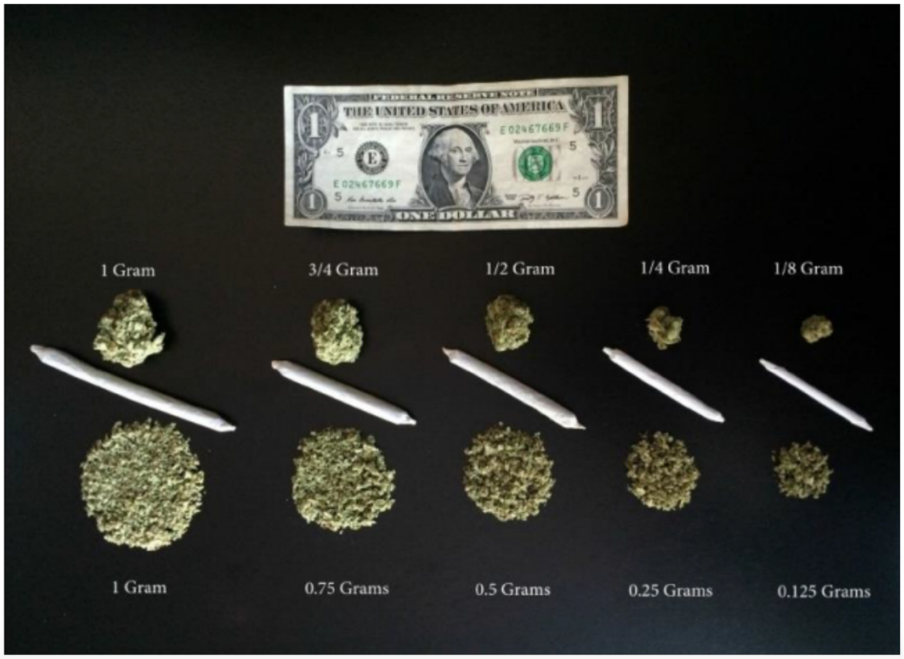

Here are the numbers that may help you:

**1/8 of a gram**	0.125
**1/4 of a gram**	0.25
**1/2 of a gram**	0.5
**1 gram**	1
**1/8 of an ounce**	3.5
**1/4 of an ounce**	7
**1/2 of an ounce**	14
**Ounce**	28

On a typical day you use cannabis, how much cannabis do you personally use (in grams)? ___________

Display This Question:
If CM = Once
Or CM = 2 to 3 days

Cmoneym. How much money do you usually spend on cannabis in a month (in dollars)? ___________

Display This Question:
If CM = 1 to 2 days per week
Or CM = 3 to 4 days per week
Or CM = 5 to 6 days per week
Or CM = Once each day
Or CM = More than once each day

Cmoneyw. How much money do you usually spend on cannabis in a week (in dollars)? ___________

Display This Question:
If CM = Once
Or CM = 2 to 3 days
Or CM = 1 to 2 days per week
Or CM = 3 to 4 days per week
Or CM = 5 to 6 days per week
Or CM = Once each day
Or CM = More than once each day

CTHC. What percentage of THC is in the cannabis you usually use?
- ☐ 0-4% (0)
- ☐ 5-9% (1)
- ☐ 10-14% (2)
- ☐ 15-19% (3)
- ☐ 20-24% (4)
- ☐ 25-29% (5)
- ☐ 30-50% (6)
- ☐ Over 50% (7)
- ☐ I don't know what THC is (996)
- ☐ I don't know my usual THC percentage (%) (998)

Display This Question:
If CM = Once
Or CM = 2 to 3 days
Or CM = 1 to 2 days per week
Or CM = 3 to 4 days per week
Or CM = 5 to 6 days per week
Or CM = Once each day
Or CM = More than once each day

CCBD. What percentage (%) of CBD is in the cannabis you usually use?
- ☐ 0-1% (0)
- ☐ 2-3% (1)
- ☐ 4-5% (2)
- ☐ 6-7% (3)

- ☐ 8-9% (4)
- ☐ 10-14% (5)
- ☐ 15-19% (6)
- ☐ 20% or more (7)
- ☐ I don't know what CBD is (996)
- ☐ I don't know my usual CBD percentage (%) (998)

Display This Question:
If CM = Once
Or CM = 2 to 3 days
Or CM = 1 to 2 days per week
Or CM = 3 to 4 days per week
Or CM = 5 to 6 days per week
Or CM = Once each day
Or CM = More than once each day

MMM. Thinking of the times you use cannabis, how often would you say you use cannabis for the following reasons?

	Never or almost never (1)	Some of the time (2)	Half of the time (3)	Most of the time (4)	Almost or almost always (5)
To forget my worries (1)	☐	☐	☐	☐	☐
Because it helps me when I feel depressed or nervous (2)	☐	☐	☐	☐	☐
To cheer me up when I am in a bad mood (3)	☐	☐	☐	☐	☐
To forget about my problems (4)	☐	☐	☐	☐	☐
Because I feel more self-confident and sure about myself (5)	☐	☐	☐	☐	☐

Display This Question:
If CM = Once
Or CM = 2 to 3 days
Or CM = 1 to 2 days per week
Or CM = 3 to 4 days per week
Or CM = 5 to 6 days per week
Or CM = Once each day
Or CM = More than once each day

Cothers1. When you use cannabis, are other people with you?
- ☐ Almost never or never (0)
- ☐ Some of the time (1)
- ☐ About half the time (2)
- ☐ A lot of the time (3)
- ☐ Most of the time or all the time (4)

Display This Question:
If Cothers1 = Some of the time
Or Cothers1 = About half the time
Or Cothers1 = A lot of the time
Or Cothers1 = Most of the time or all the time

Cothers2. When others are with you, are they also using cannabis?
- ☐ Almost never or never (0)
- ☐ Some of the time (1)
- ☐ Almost half of the time (2)
- ☐ A lot of the time (3)
- ☐ Most of the time or all the time (4)

Display This Question:
If CM = Once
Or CM = 2 to 3 days
Or CM = 1 to 2 days per week
Or CM = 3 to 4 days per week
Or CM = 5 to 6 days per week
Or CM = Once each day
Or CM = More than once each day

Cmed1. Are you currently taking cannabis for medical reasons?
- ☐ No, I **only** use cannabis for recreational purposes. (0)
- ☐ Yes, I use cannabis **only** for medical purposes. (1)
- ☐ Yes, I use cannabis for **both** medical purposes and recreational purposes.

Display This Question:
If Cmed1 = Yes, I use cannabis only for medical purposes.
Or Cmed1 = Yes, I use cannabis for both medical purposes and recreational

Cmed2 Was the cannabis you use for medicinal purposes prescribed by a medical doctor or nurse practitioner?
- ☐ No (0)
- ☐ Yes (1)

End of Block: Cannabis use

Start of Block: Alcohol Use

A1. Have you ever drank alcohol
- ☐ Yes, I drank alcohol **in the 3 months before my admission** (1)
- ☐ Yes, I have drank alcohol but **not** in the **3 months before my admission** (2)
- ☐ No, I have **never** drank alcohol (0)

Display This Question:
If A1 = Yes, I drank alcohol in the 3 months before my admission

AUDIT1. How often did you have a drink containing alcohol in the **3 months before your admission?**

- ☐ Never (0)
- ☐ Monthly or less (1)
- ☐ 2 to 4 times a month (2)
- ☐ 2 to 3 times a week (3)
- ☐ 4 or more times a week (4)

Display This Question:
If A1 = Yes, I drank alcohol in the 3 months before my admission
And AUDIT1 != Never

AUDIT2. In the **3 months before your admission**, how many drinks containing alcohol did you have on a typical day when you were drinking?

When we say a "drink", we mean:
One bottle or can of beer or a glass of draft
One glass of wine or a wine cooler
One drink or cocktail with one and a half ounces of liquor (i.e., one "shot")

- ☐ 1 or 2 (0)
- ☐ 3 or 4 (1)
- ☐ 5 or 6 (2)
- ☐ 7, 8, or 9 (3)
- ☐ 10 or more (4)

Display This Question:
If A1 = Yes, I drank alcohol in the 3 months before my admission
And AUDIT1 != Never

AUDIT3. In the **3 months before your admission**, how often did you have six or more drinks on one occasion?

- ☐ Never (0)
- ☐ Less than monthly (1)
- ☐ Monthly (2)
- ☐ Weekly (3)
- ☐ Daily or almost daily (4)

Display This Question:
If A1 = Yes, I drank alcohol in the 3 months before my admission
And AUDIT1 != Never

AUDIT4. In the **3 months before your admission**, how often did you find that you were not able to stop drinking once you had started?

- ☐ Never (0)
- ☐ Less than monthly (1)
- ☐ Monthly (2)
- ☐ Weekly (3)
- ☐ Daily or almost daily (4)

Display This Question:
If A1 = Yes, I drank alcohol in the 3 months before my admission
And AUDIT1 != Never

AUDIT5. In the **3 months before your admission**, how often did you fail to do what was normally expected of you because of drinking?

- ☐ Never (0)
- ☐ Less than monthly (1)
- ☐ Monthly (2)
- ☐ Weekly (3)
- ☐ Daily or almost daily (4)

Display This Question:
If A1 = Yes, I drank alcohol in the 3 months before my admission
And AUDIT1 != Never

AUDIT6. In the **3 months before your admission**, how often did you need a first drink in the morning to get yourself going after a heavy drinking session?

- ☐ Never (0)
- ☐ Less than monthly (1)
- ☐ Monthly (2)
- ☐ Weekly (3)
- ☐ Daily or almost daily (4)

Display This Question:
If A1 = Yes, I drank alcohol in the 3 months before my admission
And AUDIT1 != Never

AUDIT7. In the **3 months before your admission**, how often did you have a feeling of guilt or remorse after drinking?

- ☐ Never (0)
- ☐ Less than monthly (1)
- ☐ Monthly (2)
- ☐ Weekly (3)
- ☐ Daily or almost daily (4)

Display This Question:
If A1 = Yes, I drank alcohol in the 3 months before my admission
And AUDIT1 != Never

AUDIT8. In the **3 months before your admission**, how often were you unable to remember what happened the night before because of your drinking?

- ☐ Never (0)
- ☐ Less than monthly (1)
- ☐ Monthly (2)
- ☐ Weekly (3)
- ☐ Daily or almost daily (4)

Display This Question:
If A1 = Yes, I drank alcohol in the 3 months before my admission
And AUDIT1 != Never

AUDIT9 Have you or someone else been injured as a result of your drinking?
- ☐ No (0)
- ☐ Yes, but not in the last 3 months (2)
- ☐ Yes, in the last 3 months 4)

Display This Question:
If A1 = Yes, I drank alcohol in the 3 months before my admission
And AUDIT1 != Never

AUDIT10. Has a relative or friend, doctor or another health worker been concerned about your drinking or suggested you cut down?
- ☐ No (0)
- ☐ Yes, but not in the 3 months before my admission (2)
- ☐ Yes, during the 3 months before my admission (4)

Display This Question:
If C1 = Yes, I used cannabis in the 3 months before my admission
And A1 = Yes, I drank alcohol in the 3 months before my admission

Alc_Can. In the **3 months before your admission,** how often did you use alcohol and cannabis on the <u>same occasion</u> - that is, so that their effects overlapped?
- ☐ Never in my lifetime (0)
- ☐ Not in the 3 months before my admission (1)
- ☐ Monthly or less (2)
- ☐ 2 to 4 times a month (3)
- ☐ 2 to 3 times a week (4)
- ☐ 4 or more times a week (5)

Display This Question:
If A1 = Yes, I drank alcohol in the 3 months before my admission

AM. In the **4 weeks before your admission**, how often did you drink alcohol?
- ☐ Never (0)
- ☐ Once (1)
- ☐ 2 to 3 days (2)
- ☐ 1 to 2 days per week (3)
- ☐ 3 to 4 days per week (4)
- ☐ 5 to 6 days per week (5)
- ☐ Daily (6)

Display This Question:
If AM = Once
Or AM = 2 to 3 days
Or AM = 1 to 2 days per week
Or AM = 3 to 4 days per week
Or AM = 5 to 6 days per week
Or AM = Daily

AMHED. In the past **4 weeks before your admission**, how often did you have **5 or more** drinks of alcohol on the same occasion?

- ☐ Never (0)
- ☐ Once (1)
- ☐ 2 to 3 days (2)
- ☐ 1 to 2 days per week (3)
- ☐ 3 to 4 days per week (4)
- ☐ 5 to 6 days per week (5)
- ☐ Daily (6)

Display This Question:
If AM = Once
Or AM = 2 to 3 days
Or AM = 1 to 2 days per week
Or AM = 3 to 4 days per week
Or AM = 5 to 6 days per week
Or AM = Daily

AMQ. Thinking of the times you drink, how often would you say you drink for the following reasons?

	Never or almost never (1)	Some of the time (2)	Half of the time (3)	Most of the time (4)	Almost or almost always (5)
To forget my worries (1)	☐	☐	☐	☐	☐
Because it helps me when I feel depressed or nervous (2)	☐	☐	☐	☐	☐
To cheer me up when I am in a bad mood (3)	☐	☐	☐	☐	☐
To forget about my problems (4)	☐	☐	☐	☐	☐
Because I feel more self-confident and sure about myself (5)	☐	☐	☐	☐	☐

Display This Question:
If AM = Once
Or AM = 2 to 3 days
Or AM = 1 to 2 days per week
Or AM = 3 to 4 days per week
Or AM = 5 to 6 days per week
Or AM = Daily

Aothers1. Are other people with you when you drink alcohol?

- ☐ Almost never or never (0)
- ☐ Some of the time (1)
- ☐ About half the time (2)
- ☐ A lot of the time (3)
- ☐ Most of the time or all the time (4)

Display This Question:
If Aothers1 = Some of the time
Or Aothers1 = About half the time
Or Aothers1 = A lot of the time
Or Aothers1 = Most of the time or all the time

Aothers2. When others are with you, are they also drinking alcohol?

- ☐ Almost never or never (0)
- ☐ Some of the time (1)
- ☐ About half of the time (2)
- ☐ A lot of the time (3)
- ☐ Most of the time or all the time (4)

End of Block: Alcohol Use

Start of Block: Mental Health

OCHS. The statements below describe some of the feelings and behaviours of young people.

For each statement, please select the response that best describes you now or within the **3 months before your admission**. You may only select one response.

	Never or not true (1)	Sometimes or somewhat true (1)	Often or very true (2)
I am afraid of doing things in front of others (1)	☐	☐	☐
I am angry and resentful (2)	☐	☐	☐

I am anxious or on edge (3)	☐	☐	☐
I argue a lot with adults (4)	☐	☐	☐
I avoid social situations (6)	☐	☐	☐
I blame others for my own mistakes (7)			
I have trouble concentrating or paying attention (8)	☐	☐	☐
I can't stay seated when required to do so (9)	☐	☐	☐
I have had a change in appetite (10)	☐	☐	☐
I am mean to others (12)	☐	☐	☐
I destroy things belonging to others (14)	☐	☐	☐
I am easily distracted, have difficulty sticking to any activity (15)	☐	☐	☐
I don't like to be with people I don't know well (16)	☐	☐	☐

I am easily annoyed by others (17)	☐	☐	☐
I fail to finish things I start (18)	☐	☐	☐
I feel worthless or inferior (19)	☐	☐	☐
I fidget (20)	☐	☐	☐
I find it hard to stop worrying (21)	☐	☐	☐
I get anxious about meeting new people (22)	☐	☐	☐
I get back at people (23)	☐	☐	☐
I get in many fights (24)	☐	☐	☐
I get no pleasure from usual activities (25)	☐	☐	☐
I have been physically cruel to others (26)	☐	☐	☐
I have broken into someone else’s house, building or car (27)	☐	☐	☐
I have difficulty awaiting my turn in games or groups (28	☐	☐	☐

I have trouble enjoying myself (30)	☐	☐	☐
I act without stopping to think (31)	☐	☐	☐
I feel nervous with people I don’t know well (32)	☐	☐	☐
I lose my temper (33)	☐	☐	☐
I make careless mistakes (34)	☐	☐	☐
I am nervous or tense (35)	☐	☐	☐
I feel overtired or lack energy (37)	☐	☐<br>☐	☐
I run away from home (38)	☐		☐
I set fires (40)	☐	☐	☐
I stay out at night despite being told not to (41)	☐	☐	☐
I steal things from places other than home (42)	☐	☐	☐
I am too fearful or anxious (44)	☐	☐	☐

I have trouble sleeping (45)	☐	☐	☐
I cut classes or skip school (46)	☐	☐	☐
I am unhappy, sad, or depressed (47)	☐	☐	☐
I use weapons when fighting (48)	☐	☐	☐
When anxious, my mind goes blank (49)	☐	☐	☐
I worry about doing better at things (50)	☐	☐	☐
	☐	☐	☐

Psy. The next items ask about thoughts or beliefs that you could have had during the **3 months before your admission.**

	Not true (0)	Somewhat true (1)	Certainly true (2)
Some people believe that their thoughts can be read. Have other people ever read your thoughts? (1)	☐	☐	☐
Have you ever believed that you were being sent special messages through the television? (2)	☐	☐	☐

Have you ever thought that you were being followed or spied upon? (3)	☐	☐	☐
Have you ever heard voices that other people cannot hear? (4)	☐	☐	☐
Have you ever felt as though your body had been changed in some way that you could not understand? (5)	☐	☐	☐
Have you ever felt that you were under the control of some special power? (6)	☐	☐	☐
Have you ever known what another person was thinking even though that person wasn't speaking? (7)	☐	☐	☐
Do you have any special powers that other people do not have? (8)	☐	☐	☐
Have you ever seen something or someone that other people could not see? (9)	☐	☐	☐

K6. The following questions ask about how you have been feeling during the **30 days before your admission**. For each question, please pick the response that best describes how often you had this feeling.

	All of the time (4)	Most of the time (3)	Some of the time (2)	A little of the time (1)	None of the time (1)
… nervous? (1)	☐	☐	☐	☐	☐
… hopeless? (2)	☐	☐	☐	☐	☐
… restless or fidgety (3)	☐	☐	☐	☐	☐
…so depressed that nothing could cheer you up? (4)	☐	☐	☐	☐	☐
...that everything was an effort? (5)	☐	☐	☐	☐	☐
...worthless? (6)	☐	☐	☐	☐	☐

End of Block: Mental Health

Start of Block: Tobacco

T1. In the **3 months before your admission**, how often did you smoke cigarettes or cigars?

- ☐ Never in my lifetime (0)
- ☐ Not in the 3 months before my admission (1)
- ☐ Less than monthly (2)
- ☐ About once a month (3)
- ☐ 2 to 4 times a month (4)
- ☐ 2 to 3 times a week (5)
- ☐ 4 to 6 times a week (6)
- ☐ Daily (7)

T2. Electronic cigarettes **(e-cigarettes)** are battery-operated devices that look like cigarettes and create a mist which the user inhales. Some e-cigarettes contain nicotine and some do not. Other names for e-cigarettes include "vape pipes", "hookah pens", "e-hookahs", and "Juuls".

In the **3 months before your admission**, how often did you smoke **e-cigarettes**?

- ☐ Never in my lifetime (0)
- ☐ Not in the 3 months before my admission (1)
- ☐ Less than monthly (2)
- ☐ About once a month (3)
- ☐ 2 to 4 times a month (4)
- ☐ 2 to 3 times per week (5)
- ☐ 4 to 6 times per week (6)
- ☐ Daily (7)

T3. In the 3 months before your admission how often did you use cannabis and tobacco on the same occasion - that is, so their effects overlapped (including smoking cannabis mixed with tobacco, such as poppers)?

- ☐ Never in my lifetime (0)
- ☐ Not in the 3 months before my admission (1)
- ☐ Monthly or less (2)
- ☐ 2 to 4 times a month (3)
- ☐ 2 to 3 times a week (4)
- ☐ 4 or more times a week (5)

End of Block: Tobacco

Start of Block: Other Drugs

OD. The next few questions ask about use of other types of drugs. Some questions may not apply to you, but please be as honest as you can. Remember, your answers are private.

Please indicate how frequently you have used the following substances in the **3 months before your admission.**

Note: For prescription drugs, please comment on non-medical use only. This means if you used the drug without a doctor's prescription or used in a way that was not recommended by your doctor or used for the purpose of getting high.

	Never in my life	Not in the last 3 months (1)	1 or 2 times (2)	3 to 5 times (3)	6 to 9 times (4)	10 or more times (5)	I don't know what this is (996)
**K2 or Spice** (also known as K3, Blaze, Black Mamba, fake pot, IZMS) (1)	☐	☐	☐	☐	☐	☐	☐
**Cocaine** (also known as coke, blow, snow, powder, snort, etc.) (2)	☐	☐	☐	☐	☐	☐	☐
**Prescription stimulants** (Ritalin, Concerta, Dexedrine, Adderall, diet pills, etc.) (3)	☐	☐	☐	☐	☐	☐	☐
**Methamphetamine** (also known as speed, crystal meth, crank, Ice, etc.) (4)	☐	☐	☐	☐	☐	☐	☐
**Sniff or "huff" glue or other solvents** (for example, gasoline, butane, aerosols, paint thinner, nail polish remover, etc.) (5)	☐	☐	☐	☐	☐	☐	☐
**Sedatives or Tranquilizers** (such as Xanax, Valium, Ativan, also known as tranqs, benzos, xans, bars, downers) (6)	☐	☐	☐	☐	☐	☐	☐

**Hallucinogens** (LSD, acid, mushrooms, PCP, Special K, ecstasy, MDMA, molly, etc.) (7)	☐	☐	☐	☐	☐	☐	☐
**Street Opioids** (heroin, opium, etc.) (8)	☐	☐	☐	☐	☐	☐	☐
**Prescribed opioids or pain relief pills** (fentanyl, oxycodone [OxyContin, Percocet], hydrocodone [Vicodin], methadone, buprenorphine, Tylenol #3, codeine, etc.). *We do not mean regular Tylenol, Advil, or Aspirin that anyone can buy in a drugstore.* (9)	☐	☐	☐	☐	☐	☐	☐
**Steroids**, body builders/performance builders (e.g., clenbuterol "clen", anavar, trenbolone "tren", testosterone and other androgens, durabolin, growth hormones, etc.) (10)	☐	☐	☐	☐	☐	☐	☐

End of Block: Other Drugs

Start of Block: Service Use

MHS1. In the **3 months <u>before</u> your admission**, did you see or talk to anyone from the following places about any concerns you may have had about your **mental health**? This does <u>not</u> include your current hospital visit/admission.

	Did not see/talk to anyone here (0)	1 or 2 times (1)	Once a month (2)	2 or 3 times a month (3)	Once a week or more (4)
A doctor's office (you may have talked to a nurse or a doctor) (1)	☐	☐	☐	☐	☐
A counsellor’s office outside of school, such as a psychiatrist, psychologist, social worker or other type of counsellor (2)	☐	☐	☐	☐	☐
A nurse or counsellor's office at school (3)	☐	☐	☐	☐	☐
An agency that provides mental health or addictions services for youth (4)	☐	☐	☐	☐	☐
A day-hospital program (6)	☐	☐	☐	☐	☐
An urgent care clinic or emergency room (5)	☐	☐	☐	☐	☐
A mental health assessment unit (like a psychiatric emergency department) (8)	☐	☐	☐	☐	☐
An overnight hospital program (inpatient admission) (7)	☐	☐	☐	☐	☐

MHS2. In the **3 months <u>before</u> your admission**, did you see or talk to anyone from the following places about any concerns you may have had about your **substance use**? *Please select all that apply.*

- ☐ A doctor's office (you may have talked to a nurse or a doctor) (1)
- ☐ A counsellor's office **outside of school**, such as a psychiatrist, psychologist, social worker or other type of counsellor (2)
- ☐ A nurse or counsellor's office **at school** (3)
- ☐ An agency that provides mental health or addictions services for youth (4)
- ☐ A day-hospital program (5)
- ☐ An urgent care clinic or emergency room (6)
- ☐ A mental health assessment unit (like a psychiatric emergency department) (7)
- ☐ An overnight hospital program (inpatient admission) (8)
- ☐ Did not see or talk to anyone about substance use (9)

End of Block: Service Use

Start of Block: Demographics

D4. Which of the following best describes your background? **Please select all that apply.**

Are you...?

- ☐ White (e.g., British, French, Italian, Portuguese, Ukrainian, Russian, Israeli) (1)
- ☐ Chinese (2)
- ☐ South Asian (e.g., East Indian, Pakistani, Bangladeshi, Sri Lankan) (3)
- ☐ Black (African, Caribbean, North American) (4)
- ☐ Aboriginal (First Nations, Inuit, Métis, non-status Indian) (5)
- ☐ Filipino (6)
- ☐ Latin American, Central American, South American (e.g., Mexican, Brazilian, Chilean, Guatemalan, Venezuelan, Colombian, Argentinian, Salvadoran, Costa Rican) (7)
- ☐ Southeast Asian (e.g., Vietnamese, Cambodian, Indonesian, Malaysian, Laotian) (8)
- ☐ West Asian or Arab (e.g., Egyptian, Saudi Arabian, Syrian, Iranian, Iraqi, Lebanese, Afghan, Palestinian) (9)
- ☐ Korean (10)
- ☐ Japanese (11)
- ☐ Not sure (998)

D5. How long have you lived in Canada?
- ☐ All my life (1)
- ☐ 2 years or less (2)
- ☐ 3-5 years (3)
- ☐ 6-10 years (4)
- ☐ 11 years or longer (5)
- ☐ Not sure (998)

D6. Were your parents born in Canada?
- ☐ Two (or more) parents born in Canada (0)
- ☐ One parent born in Canada (1)
- ☐ No parent born in Canada (2)
- ☐ Not sure (998)

D8. At the top of the ladder are the people who are best off - they have the most money, the highest amount of schooling and the jobs that bring the most respect. At the bottom of the ladder are the people who are worst off - they have the least money, little or no education, no job or jobs that no one wants or respects.

- ☐ 1 (Worst off) (1)
- ☐ 2 (2)
- ☐ 3 (3)
- ☐ 4 (4)
- ☐ 5 (5)
- ☐ 6 (6)
- ☐ 7 (7)
- ☐ 8 (8)
- ☐ 9 (9)
- ☐ 10 (Best off) (10)

End of Block: Demographics (Extended)

Start of Block: Final

You have now reached the end of the survey. Click the 'next' button to submit your answers

End of Block: Final
