# Supplementary material for: The CAMP study: feasibility and clinical correlates of standardized assessments of substance use in a youth psychiatric inpatient sample
Source: Child Adolesc Psychiatry Ment Health. 2021 Sep 13;15:48. doi: 10.1186/s13034-021-00403-4 (PMC8439003; doi:10.1186/s13034-021-00403-4)
Supplement: Supplementary file 3 — Additional file 3. Staff survey. [file 13034_2021_403_MOESM3_ESM.pdf]

Assessing and Addressing Substance Use on the Inpatient Child and Youth Mental Health Unit:

Frontline Staff Questionnaire

1. **What is your role on the unit?**

- ○ Full Time or Part Time Nurse (1)
- ○ Full Time or Part Time Child and Youth Worker (2)

---

2. **How long have you worked on the unit?**

- ○ <6 months (1)
- ○ <1 year (2)
- ○ 1-2 years (3)
- ○ 3-4 years (4)
- ○ 5+ years (5)

---

3. **Have you received any specialized substance use training?**

- ○ No (0)
- ○ Yes - What was it? (1) ____

4. How **confident** are you in your **knowledge** on how the following substances may impact youth on the unit?

	Not at all confident (0)	Slightly confident (1)	Somewhat confident (2)	Fairly confident (3)	Completely confident (4)
Alcohol (1)	○	○	○	○	○
Cannabis (2)	○	○	○	○	○
Tobacco/Nicotine (3)	○	○	○	○	○
Opioids (non-medical) (4)	○	○	○	○	○
Benzodiazepines/sedatives (non-medical) (5)	○	○	○	○	○
ADHD medications (non-medical) (6)	○	○	○	○	○
Other illicit drugs (hallucinogens, cocaine, solvents, methamphetamine) (7)	○	○	○	○	○

4.B Other comments regarding **knowledge** on how substances may impact the youth on the unit:

____

____

____

____

____

5. How **confident** are you in your **ability to identify and respond to withdrawal symptoms** for the following substances? Note: *responding* includes pharmacological (nursing only), psychotherapeutic, and other comfort and self-care strategies.

	Not at all confident (0)	Slightly confident (1)	Somewhat confident (2)	Fairly confident (3)	Completely confident (4)
Alcohol (1)	○	○	○	○	○
Cannabis (2)	○	○	○	○	○
Tobacco/Nicotine (3)	○	○	○	○	○
Opioids (non-medical use) (4)	○	○	○	○	○
Benzodiazepines/sedatives (non-medical use) (5)	○	○	○	○	○
ADHD medications (non-medical use) (6)	○	○	○	○	○
Other illicit drugs (hallucinogens, cocaine, solvents, methamphetamine) (7)	○	○	○	○	○

5.B Other comments related to **identifying and responding to withdrawal:**

____

____

____

____

____

6. In your role, **what do you currently do** to assess and/or address substance use among youth on the unit?

7. What do you think **could be done differently** (if anything)?

8. **What may get in the way** of comprehensive substance use assessments on the unit? Please select all barriers that apply.

- ☐ Time pressures (1)
- ☐ Screening for substance use is the function of other health services (2)
- ☐ Do not know what to do if youth screen positive while on the unit (3)
- ☐ Unfamiliar with treatment resources in the community (4)
- ☐ Uncertainty regarding the effectiveness of available treatments (5)
- ☐ Youth do not often tell the truth about their substance use (6)
- ☐ Documentation of substance use problems in the medical record may adversely affect youth (7)
- ☐ Do not want youth to worry about who will be informed about their substance use (8)
- ☐ Personally uncomfortable talking about substance use with youth (9)
- ☐ Lack of training (10)
- ☐ Lack of space and privacy for conversations (11)
- ☐ Lack of funds to make system changes (12)
- ☐ I do not foresee any barriers to changing screening procedures (13)
- ☐ Other barriers not listed or comments: (14) ____

9. What may **facilitate** comprehensive substance use assessment on the unit? Please select all facilitators that apply.

- ☐ Adding specific questions to the electronic medical record (1)
- ☐ Adding a space in the Kardex to flag substance use concerns (2)
- ☐ Training on how to ask questions related to substance use (3)
- ☐ Training on pharmacological options for addressing substance use (4)
- ☐ Training on how to deliver psychoeducation (5)
- ☐ Training on psychotherapeutic approaches for addressing substance use (6)
- ☐ Other facilitators not listed or comments: (7)

____

10. How **confident** would you be in **delivering brief psycho-education** on the following substances?

	Not at all confident (0)	Slightly confident (1)	Somewhat confident (2)	Fairly confident (3)	Completely confident (4)
Alcohol (1)	○	○	○	○	○
Cannabis (2)	○	○	○	○	○
Tobacco/Nicotine (3)	○	○	○	○	○
Opioids (non-medical) (4)	○	○	○	○	○
Benzodiazepines/sedatives (non-medical) (5)	○	○	○	○	○
ADHD medications (non-medical) (6)	○	○	○	○	○
Other illicit drugs (hallucinogens, cocaine, solvents, methamphetamine) (7)	○	○	○	○	○

10.B Other comments regarding **confidence in delivering brief psycho-education**:

____

____

____

____

____

11. How **confident** would you be **delivering a brief motivational intervention for substance use?**

- ◯ Not at all confident (0)
- ◯ Slightly confident (1)
- ◯ Somewhat confident (2)
- ◯ Fairly confident (3)
- ◯ Completely confident (4)

---

12. What are your thoughts on including substance use (both occasional and regular) in the patient conceptualization and treatment plan on the unit? How do you think this could be facilitated? What may get in the way?

____

____

____

---

13. What **education or training** would you like regarding substance use (if any)?

____

____

____

---

14. **Other thoughts about assessing and addressing substance use on the unit:**

____

____

____
