# Supplementary material for: The CAMP study: feasibility and clinical correlates of standardized assessments of substance use in a youth psychiatric inpatient sample
Source: Child Adolesc Psychiatry Ment Health. 2021 Sep 13;15:48. doi: 10.1186/s13034-021-00403-4 (PMC8439003; doi:10.1186/s13034-021-00403-4)
Supplement: Supplementary file 4 — Additional file 4. Reporting guidelines. [file 13034_2021_403_MOESM4_ESM.docx]

**The RECORD statement – checklist of items, extended from the STROBE statement, that should be reported in observational studies using routinely collected health data.**

|  | **Item No.** | **STROBE items** | **Location in manuscript where items are reported** | **RECORD items** | **Location in manuscript where items are reported** |
| --- | --- | --- | --- | --- | --- |
| **Title and abstract** | | | | | |
|  | 1 | (a) Indicate the study’s design with a commonly used term in the title or the abstract (b) Provide in the abstract an informative and balanced summary of what was done and what was found | (a) Yes, Title Page: Stated observational study in title  (b) Yes Page 2: Abstract methods discuss summary | RECORD 1.1: The type of data used should be specified in the title or abstract. When possible, the name of the databases used should be included.  RECORD 1.2: If applicable, the geographic region and timeframe within which the study took place should be reported in the title or abstract.  RECORD 1.3: If linkage between databases was conducted for the study, this should be clearly stated in the title or abstract. | - 1. In abstract – names of databases not included due to word count limits   2. In abstract – methods   3. N/A for this paper |
| **Introduction** | | | | | |
| Background rationale | 2 | Explain the scientific background and rationale for the investigation being reported | Yes (Pages 3-4) |  |  |
| Objectives | 3 | State specific objectives, including any prespecified hypotheses | Yes (Page 4) |  |  |
| **Methods** | | | | | |
| Study Design | 4 | Present key elements of study design early in the paper | Yes (Page 4): Under Design and Setting |  |  |
| Setting | 5 | Describe the setting, locations, and relevant dates, including periods of recruitment, exposure, follow-up, and data collection | Yes (Pages 5):  under Design and Setting and under Participants |  |  |
| Participants | 6 | *(a) Cohort study* - Give the eligibility criteria, and the sources and methods of selection of participants. Describe methods of follow-up  *Case-control study* - Give the eligibility criteria, and the sources and methods of case ascertainment and control selection. Give the rationale for the choice of cases and controls  *Cross-sectional study* - Give the eligibility criteria, and the sources and methods of selection of participants  *(b) Cohort study* - For matched studies, give matching criteria and number of exposed and unexposed  *Case-control study* - For matched studies, give matching criteria and the number of controls per case | (a) Yes (Page 5-8)  Page 5: Sources of selection under Design and Setting  Page 6: eligibility criteria methods of selection under Sample  Page 8: Under recruitment and Data Collection  Page 11: under results recruitment and data collection strategy  (b) N/A | RECORD 6.1: The methods of study population selection (such as codes or algorithms used to identify subjects) should be listed in detail. If this is not possible, an explanation should be provided.  RECORD 6.2: Any validation studies of the codes or algorithms used to select the population should be referenced. If validation was conducted for this study and not published elsewhere, detailed methods and results should be provided.  RECORD 6.3: If the study involved linkage of databases, consider use of a flow diagram or other graphical display to demonstrate the data linkage process, including the number of individuals with linked data at each stage. | 6.1 and 6.2– see Youth Chart Reviews methods section and Additional File 2 for full list of codes  and extraction. |
| Variables | 7 | Clearly define all outcomes, exposures, predictors, potential confounders, and effect modifiers. Give diagnostic criteria, if applicable. | Yes (Page 6, Table 1, and Page 9) | RECORD 7.1: A complete list of codes and algorithms used to classify exposures, outcomes, confounders, and effect modifiers should be provided. If these cannot be reported, an explanation should be provided. | Specific details about location in MEDITECH or Sovera provided in Additional File 2 and all administrative codes provided. |
| Data sources/ measurement | 8 | For each variable of interest, give sources of data and details of methods of assessment (measurement).  Describe comparability of assessment methods if there is more than one group | Yes (Page 6 and 7) – Under Measures with further details provided in Table 1 |  |  |
| Bias | 9 | Describe any efforts to address potential sources of bias | Yes.  Some considerations under “Ethics”, Page 8-9 and lines 387-393 |  |  |
| Study size | 10 | Explain how the study size was arrived at | Yes (Additional File 5) |  |  |
| Quantitative variables | 11 | Explain how quantitative variables were handled in the analyses. If applicable, describe which groupings were chosen, and why | Yes (Pages 9-10) Under Statistical Analysis |  |  |
| Statistical methods | 12 | (a) Describe all statistical methods, including those used to control for confounding  (b) Describe any methods used to examine subgroups and interactions  (c) Explain how missing data were addressed  (d) *Cohort study* - If applicable, explain how loss to follow-up was addressed  *Case-control study* - If applicable, explain how matching of cases and controls was addressed  *Cross-sectional study* - If applicable, describe analytical methods taking account of sampling strategy  (e) Describe any sensitivity analyses | (a) Yes (Page 9-10) Under “Statistical Analysis”  (b) Yes (Page 10)  (c) Yes (Page 10)  (d) Yes (page 10) |  |  |
| Data access and cleaning methods |  | .. |  | RECORD 12.1: Authors should describe the extent to which the investigators had access to the database population used to create the study population.  RECORD 12.2: Authors should provide information on the data cleaning methods used in the study. | Details available in Additional File 2 . |
| Linkage |  | .. |  | RECORD 12.3: State whether the study included person-level, institutional-level, or other data linkage across two or more databases. The methods of linkage and methods of linkage quality evaluation should be provided. | 12.3 page 11 – under recruitment and data collection and in content of chart reviews |
| **Results** | | | | | |
| Participants | 13 | (a) Report the numbers of individuals at each stage of the study (*e.g.*, numbers potentially eligible, examined for eligibility, confirmed eligible, included in the study, completing follow-up, and analysed)  (b) Give reasons for non-participation at each stage.  (c) Consider use of a flow diagram | (a) Yes (Page 10 and 14) – under response rate and retention and Figure 1  (b) Yes (Page 10 and 14) – under response rate and retention and Figure 1 | RECORD 13.1: Describe in detail the selection of the persons included in the study (*i.e.,* study population selection) including filtering based on data quality, data availability and linkage. The selection of included persons can be described in the text and/or by means of the study flow diagram. | Page 5-6 – design and settings, participants, recruitment and data collection  Figure 1 – CONSORT flow diagram |
| Descriptive data | 14 | (a) Give characteristics of study participants (*e.g.*, demographic, clinical, social) and information on exposures and potential confounders  (b) Indicate the number of participants with missing data for each variable of interest  (c) *Cohort study* - summarise follow-up time (*e.g.*, average and total amount) | (a) Yes (Page 11-12 and Table 2)  b) Yes – Page 10 under results Response Rates and Retention  c) Yes – Page 10 under Response Rates and Retention |  |  |
| Outcome data | 15 | *Cohort study* - Report numbers of outcome events or summary measures over time  *Case-control study* - Report numbers in each exposure category, or summary measures of exposure  *Cross-sectional study* - Report numbers of outcome events or summary measures | Yes, Figure 1 and page 10-11 |  |  |
| Main results | 16 | (a) Give unadjusted estimates and, if applicable, confounder-adjusted estimates and their precision (e.g., 95% confidence interval). Make clear which confounders were adjusted for and why they were included  (b) Report category boundaries when continuous variables were categorized  (c) If relevant, consider translating estimates of relative risk into absolute risk for a meaningful time period | (a) Yes – Table 4 and under most sections of results  (b) Yes - cut-offs identified in all tables and most sections of results  (c) NR |  |  |
| Other analyses | 17 | Report other analyses done—e.g., analyses of subgroups and interactions, and sensitivity analyses | Yes – Page 14 under clinical correlates of substance use and Table 4 as well as Additional File 5 for more detailed results. |  |  |
| **Discussion** | | | | | |
| Key results | 18 | Summarise key results with reference to study objectives | Yes – Page 15-16 under discussions, paragraphs 1-3 |  |  |
| Limitations | 19 | Discuss limitations of the study, taking into account sources of potential bias or imprecision. Discuss both direction and magnitude of any potential bias | Yes – Page 17-18 | RECORD 19.1: Discuss the implications of using data that were not created or collected to answer the specific research question(s). Include discussion of misclassification bias, unmeasured confounding, missing data, and changing eligibility over time, as they pertain to the study being reported. | Yes – Page 17-18 |
| Interpretation | 20 | Give a cautious overall interpretation of results considering objectives, limitations, multiplicity of analyses, results from similar studies, and other relevant evidence | Yes – Page 18-19 |  |  |
| Generalisability | 21 | Discuss the generalisability (external validity) of the study results | Yes – Page 17-18 |  |  |
| **Other Information** | | | | | |
| Funding | 22 | Give the source of funding and the role of the funders for the present study and, if applicable, for the original study on which the present article is based | Yes, page 21 |  |  |
| Accessibility of protocol, raw data, and programming code |  |  | Complete questionnaires and chart review data and codes are provided in Additional Files. Cannot provide open data access due to ethics/privacy constraints. | RECORD 22.1: Authors should provide information on how to access any supplemental information such as the study protocol, raw data, or programming code. | This paper provides specific details regarding the study protocol with extensive Additional Files. Author contact information provided for further clarification or information.  materials |

*Reference: Benchimol EI, Smeeth L, Guttmann A, Harron K, Moher D, Petersen I, Sørensen HT, von Elm E, Langan SM, the RECORD Working Committee. The REporting of studies Conducted using Observational Routinely-collected health Data (RECORD) Statement. *PLoS Medicine* 2015; in press.

*Checklist is protected under Creative Commons Attribution ([CC BY](http://creativecommons.org/licenses/by/4.0/)) license.
