# Supplementary material for: The CAMP study: feasibility and clinical correlates of standardized assessments of substance use in a youth psychiatric inpatient sample
Source: Child Adolesc Psychiatry Ment Health. 2021 Sep 13;15:48. doi: 10.1186/s13034-021-00403-4 (PMC8439003; doi:10.1186/s13034-021-00403-4)

CAMP Additional File 5: Detailed Methods & Results

1. Sample size calculations and hypothesized prevalence estimates
2. Follow-up missingness analysis
3. Substance use disaggregated by sex and gender
4. Substance Use Disorder scores and exploration
5. Substance use and clinical severity
6. Substance use and clinical complexity
7. Substance use and service use
8. Staff survey qualitative methods and written summary of results

1. Pilot sample size calculations

Pilots do not require sample size calculations and do not need to be adequately powered for a particular effect sizes.[1] Therefore, we aimed to recruit a sample large enough to provide enough information to guide the methods and processes for a future full-scale study. Firstly, we considered prevalence of cannabis and alcohol use. Among a general population sample of grade 7-12 students in Ontario during the 2016/2017 academic year, 12.1% and 16.9% endorsed using cannabis and engaging in heavy drinking in the past month respectively.[2] Cannabis and heavy drinking are even more common among individuals with mental health concerns.[3] Of note, prevalence of cannabis use has been increasing among Canadian youth in recent years.[4] Further, using the School Mental Health Surveys - a similar representative sample of grade 6-12 students across Ontario during the 2014/2015 academic year - prevalence estimates of occasional or regular cannabis use and heavy drinking were, respectively, 1.7 and 1.5 times more compared to the general population among youth endorsing high levels of internalizing and externalizing symptomatology (i.e., 1 standard deviation or greater than the general sample). Given general population estimates, we anticipated that among 100 youth, a minimum of 20 and 25 youth would endorse at least monthly cannabis use and heavy drinking respectively. We anticipated these numbers would be higher given recent increasing trends in youth cannabis use alongside national cannabis legalization and the acuity of the sample. Secondly, we considered precision around feasibility estimates, basing success of the pilot around the 95% confidence interval (CI) of recruitment and follow-up.[1]

Of note, using G*Power software,[5] it was determined that a total sample size of 1230 will be required for the full study to analyze associations between substance use and clinical characteristics and service use, assuming a small difference in means (d=0.2), using a 2-sided independent t-test, with an a=0.05, B=0.2, and 4:1 difference in group membership (i.e. if n_{nouse}=80% and n_{use}=20%, the allocation ratio n_{nouse}/ n_{use}=4). Estimates of effect sizes come from a recent meta-analysis of the general population which found point estimates between cannabis use and developing depression, anxiety and suicidal ideation to be 1.37, 1.18, and 1.5 respectively.[6] Therefore, our study represents less than 10% of the target sample size for an adequately powered analysis to find a small significantly difference between youth who use cannabis versus those that do not, although this may be an underestimation of prevalence of youth in this sample and effect estimates in the target acute population.

1. Thabane L, Ma J, Chu R, Cheng J, Ismaila A, Rios LP, et al. A tutorial on pilot studies: the what, why and how. BMC Med Res Methodol. 2010;10(1):1.
2. Boak A, Hamilton HA, Adlaf EM, Mann RE. Drug use among Ontario students, 1977-2017: Detailed Findings from the Ontario Student Drug Use and Health Survey (OSDUHS) (CAMH Research Document Series No. 46). Toronto, ON: Centre for Addiction and Mental Health; 2017.
3. Rush B, Urbanoski K, Bassani D, Castel S, Wild TC, Strike C, et al. Prevalence of co-occurring substance use and other mental disorders in the Canadian population. Can J Psychiatry. 2008;53(12):800-9.
4. Zuckermann AME, Battista K, de Groh M, Jiang Y, Leatherdale ST. Prelegalisation patterns and trends of cannabis use among Canadian youth: results from the COMPASS prospective cohort study. BMJ Open. 2019;9(3):e026515.
5. Faul F, Erdfelder E, Lang A-G, Buchner A. G* Power 3: A flexible statistical power analysis program for the social, behavioral, and biomedical sciences. Behavior Research Methods. 2007;39(2):175-191.
6. Gobbi G, Atkin T, Zytynski T, Wang S, Askari S, Boruff J, et al. Association of cannabis use in adolescence and risk of depression, anxiety, and suicidality in young adulthood: a systematic review and meta-analysis. JAMA Psychiatry. 2019;76(4):426-34.

2. Follow-up missingness analysis

Univariable logistic regressions predicting 6-month follow-up missingness (missing=1, completed=0).

	OR (SE)	P value
Mental Health Severity		
Total OCHS-EBS	1.00 (0.015)	0.989
Internalizing OCHS-EBS	0.948 (0.030)	0.079
Externalizing OCHS-EBS	1.03 (0.022)	0.180
Psychological distress (K6)	0.913 (0.043)	0.034
Psychosis symptoms	1.05 (0.047)	0.302
Nonsuicidal self-injury	0.605 (0.277)	0.070
Suicide attempt	0.775 (0.267)	0.388
Aggression	1.197 (0.239)	0.451
Mental Health Complexity		
OCHS diagnosis count (self-reported complexity)	0.994 (0.12)	0.960
Physician reported diagnoses count (physician-reported complexity)	1.066 (0.134)	0.635
Hospital Service Use History		
Prior psychiatric inpatient admission	0.690 (0.411)	0.368
Prior psychiatric ED visit	0.420 (0.414)	0.036
Substance Use Frequency		
Cannabis	0.914 (0.180)	0.617
Alcohol	0.755 (0.174)	0.108
Tobacco Cigarettes	1.021 (0.183)	0.908
E-cigarettes	0.928 (0.178)	0.676
Prescription Drug Misuse	0.796 (0.237)	0.337
Other Drug use	0.760 (0.264)	0.299
Demographics		
Female Gender (ref male)	0.684 (0.451)	0.401
Transgender or Gender Diverse (ref male)	0.353 (0.945)	0.270
Female Sex	0.606 (0.532)	0.346
Age	0.924 (0.166)	0.632
Self-Reported Social Status	0.971 (0.121)	0.808
White Race/ethnicity	0.579 (0.453)	0.228

3. Substance use disaggregated by sex and gender

**Substance**	**Time Period** (ref to index)	**Total Sample** (n=100)	**Female Sex** (n=82)	**Male Sex** (n=18)	**Transgender or Gender Diverse** (n=19)	**Cisgender** (n=81)
Alcohol	lifetime	73%	61 (74%)	12 (67%)	16 (84%)	57 (70%)
	3 months	51%	42 (51%)	9 (50%)	12 (63%)	39 (48%)
	HED past month	29%	24 (29.3%)	5 (27.8%)	4 (21.1%)	25 (30.9%)
	AUDIT>=8 (for those who used)	23 (47%)	21 (52.5%)	2 (22.2%)	5 (45.5%)	18 (47.4%)
Cannabis	lifetime	66%	53 (65%)	13 (72%)	13 (68%)	53 (65%)
	3 months	50%	39 (48%)	11 (61%)	9 (47%)	41 (51%)
	daily past month	18%	14 (17.1%)	4 (22.2%)	3 (15.8%)	15 (18.5%)
	CUDIT>=8 (of those how have used)	32 (64%)	24 (61.5%)	8 (72.7%)	6 (66.7%)	26 (63.4%)
Tobacco	lifetime	47%	39 (48%)	8 (44%)	8 (42%)	39 (48%)
	3 months	33%	29 (35%)	4 (22%)	7 (37%)	26 (32%)
	daily past month	14%	14 (17.1%)	0	4 (21.2%)	10 (12.3%)
E-cigarettes	lifetime	42%	34 (42%)	8 (44%)	7 (37%)	35 (43%)
	3 months	34%	29 (35%)	5 (28%)	5 (26%)	29 (36%)
	daily past month	14%	13 (15.9%)	1 (5.6%)	2 (10.5%)	12 (14.8%)
Prescription Opioids	lifetime	22%	17 (21%)	5 (28%)	4 (21%)	18 (23%)
	3 months	18%	14 (18%)	4 (22%)	4 (21%)	14 (18%)
Any Alcohol, Cannabis, or Tobacco	3 months	66%	52 (63%)	14 (78%)	14 (74%)	53 (64%)
Any Prescription drug	3 months	24%	18 (22%)	6 (33%)	4 (21%)	20 (25%)
Any Illicit substance use	3 months	17%	14 (17%)	3 (17%)	2 (11%)	15 (19%)
Any substance use	3 months	69%	55 (67%)	14 (78%)	15 (79%)	54 67%)

4. Substance Use Disorder scores and exploration

Figure 4A. Baseline Cannabis Use Disorder Identification Test Revised (CUDIT) Scores

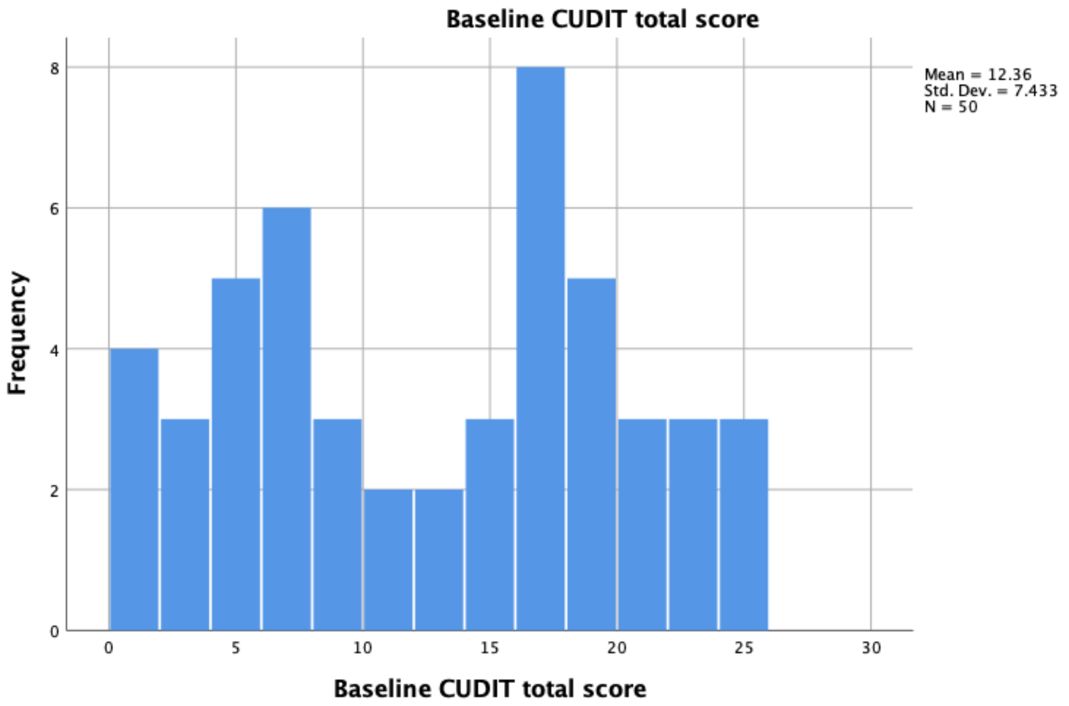

Table 4.1. Correlations between CUDIT scores and other cannabis and substance use variables.

	Kendall's tau_b	Baseline CUDIT total score
Baseline frequency of cannabis use	Correlation Coefficient	.542**
	Sig. (2-tailed)	.000
	N	50
How old were you the first time you used cannabis?	Correlation Coefficient	-.037
	Sig. (2-tailed)	.726
	N	50
What percentage of THC is in the cannabis you usually use?	Correlation Coefficient	.108
	Sig. (2-tailed)	.544
	N	19
What percentage (%) of CBD is in the cannabis you usually use?	Correlation Coefficient	.025
	Sig. (2-tailed)	.909
	N	14
Baseline Marijuana Coping Motives Score	Correlation Coefficient	.250*
	Sig. (2-tailed)	.021

	N	45
Coping Motive Item 1: To forget my worries	Correlation Coefficient	.248*
	Sig. (2-tailed)	.032
	N	45
Coping Motive Item 2: Because it helps me when I feel depressed or nervous	Correlation Coefficient	.252*
	Sig. (2-tailed)	.030
	N	45
Coping Motive Item 3: To cheer me up when I am in a bad mood	Correlation Coefficient	.380**
	Sig. (2-tailed)	.001
	N	45
Coping Motive Item 4: To forget about my problems	Correlation Coefficient	.283*
	Sig. (2-tailed)	.015
	N	45
Coping Motive Item 5: Because I feel more self-confident and sure about myself	Correlation Coefficient	.097
	Sig. (2-tailed)	.402
	N	45
Baseline frequency of alcohol use	Correlation Coefficient	.194
	Sig. (2-tailed)	.075
	N	50
Baseline past month any alcohol use or binge drinking	Correlation Coefficient	.106
	Sig. (2-tailed)	.349
	N	50
Baseline Alcohol Coping Motives Score	Correlation Coefficient	.003
	Sig. (2-tailed)	.981
	N	40
Tobacco use at Baseline	Correlation Coefficient	.131
	Sig. (2-tailed)	.232
	N	50
E-cigarette use at Baseline	Correlation Coefficient	.121
	Sig. (2-tailed)	.272
	N	50
Any prescription drug misuse in 3months prior to admission	Correlation Coefficient	.151
	Sig. (2-tailed)	.208
	N	50
Any illicit substance use in 3months prior to admission	Correlation Coefficient	.182
	Sig. (2-tailed)	.129
	N	50

Predicting CUDIT Scores

Using forward selection based on bivariate correlations, linear regression revealed that questions regarding frequency of cannabis use and coping motives explain 60.4% of the variance (adjusted R^2 0.585). This model fit better than frequency of cannabis alone (adjusted R^2 0.501). Adding frequency of using with others did not increase adjusted variance explained (adjusted R^2 0.582). Among those who also used tobacco, frequency of co-use was not significant after adjusting for frequency and motives.

Figure 4B. Baseline Alcohol Use Disorder Identification Test (AUDIT) Scores

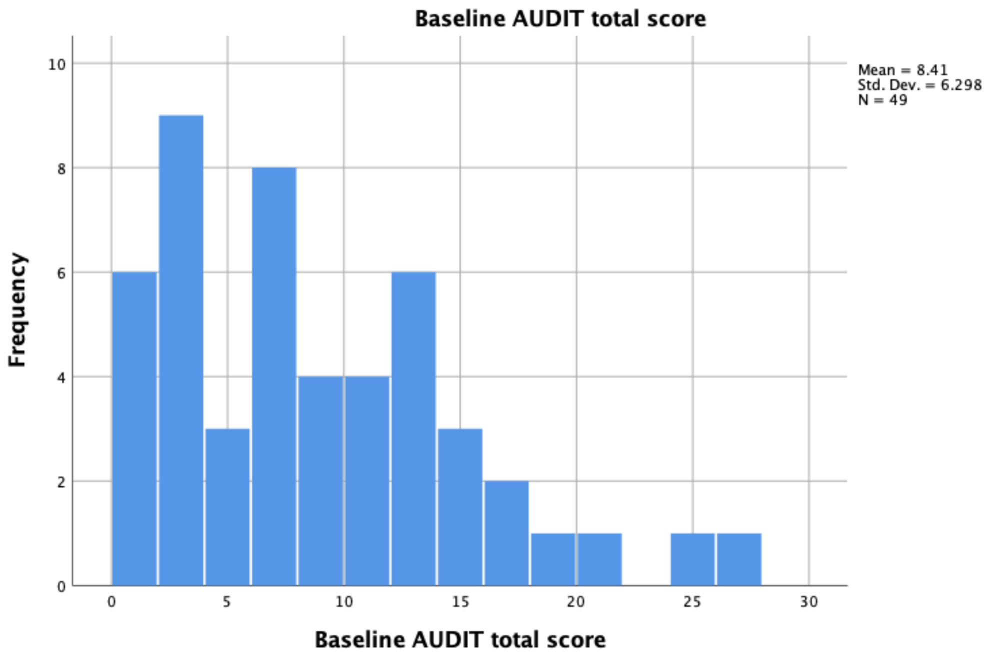

Table 4.3. Correlations between AUDIT scores and other alcohol and substance use variables.

	Kendall's tau_b	Baseline AUDIT total score
Baseline frequency of alcohol use	Correlation Coefficient	.537**
	Sig. (2-tailed)	.000
	N	49
Baseline past month any alcohol use or binge drinking	Correlation Coefficient	.551**
	Sig. (2-tailed)	.000
	N	49

Baseline Alcohol Coping Motives Score	Correlation Coefficient	.541**
	Sig. (2-tailed)	000
	N	44
Motives Item 1. To forget my worries	Correlation Coefficient	.451**
	Sig. (2-tailed)	.000
	N	44
Motives Item 2. Because it helps me when I feel depressed or nervous	Correlation Coefficient	.475**
	Sig. (2-tailed)	.000
	N	44
Motives Item 3. To cheer me up when I am in a bad mood	Correlation Coefficient	.481**
	Sig. (2-tailed)	.000
	N	44
Motives Item 4. To forget about my problems	Correlation Coefficient	.457**
	Sig. (2-tailed)	.000
	N	44
Motives Item 5. Because I feel more self-confident and sure about myself	Correlation Coefficient	.501**
	Sig. (2-tailed)	.000
	N	44
Are other people with you when you drink alcohol?	Correlation Coefficient	-.209
	Sig. (2-tailed)	.083
	N	44
Baseline frequency of cannabis use	Correlation Coefficient	.204
	Sig. (2-tailed)	.054
	N	49
How often did you use alcohol and cannabis on the same occasion?	Correlation Coefficient	.337**
	Sig. (2-tailed)	.008
	N	38
Tobacco use at Baseline	Correlation Coefficient	.424**
	Sig. (2-tailed)	.000
	N	49
E-cigarette use at Baseline	Correlation Coefficient	.295**
	Sig. (2-tailed)	.008
	N	49
Any prescription drug misuse in 3 months prior to admission	Correlation Coefficient	.212
	Sig. (2-tailed)	.082
	N	49
Any illicit substance use in 3 months prior to admission	Correlation Coefficient	.234
	Sig. (2-tailed)	.055
	N	49

Predicting AUDIT scores

Using forward selection based on bivariate correlations, linear regression revealed that questions regarding past month drinking (split into none, any but no HED, and HED) alongside coping motives for drinking explain 57.7% of the variance (adjusted R^2 0.557). This model fit better than past month drinking/HED alone (R^2 0.379) or coping motives alone (R^2 0.407). Adding frequency of tobacco use to the regression did not improve the model (adjusted R^2 0.553). Among those who also used cannabis, frequency of co-use slightly improved prediction to 59.9% (adjusted R^2 0.564). Of note, frequency of solitary use did not improve model fit.

5. Substance use and clinical severity

Part A: Substance use and clinical severity at index

Indicators of clinical psychiatric severity were operationalized based on clinician-reports from chart reviews and based on youth self-reported surveys.

Severity indicators from charts used for correlation analyses included:

1. Suicide plan (1) and attempt (2) compared to ideation or none (0). Response options of none and suicidal ideation were collapsed as only 1 patient indicated no suicide related thoughts or behaviours.
2. Self-harm previous (1) and current (2) versus none (0).
3. Aggression categories were collapsed based on overlap and frequencies into aggressive threats or aggression without harm (1), aggression resulting in property damage or harm to others (2), and none (0). Some youth fell into multiple domains and the highest level of aggression reported was coded in this indicator.

Several severity indicators from chart reviews were not included in subsequent analyses due to low frequency counts and/or missing data/lack of clarity, including:

- Patient (19 missing), parent (33 missing), and clinician ($n<10$) reported psychosis, bizarre, or disorganized behaviour documented in the medical record.
- Homicidal ideation, plans, or attempts ($n<10$) documented in medical record.
- Use of restraints ($n<10$) or an egregious behaviour analyses ($n<10$) while on the unit documented in medical record.

Severity indicators from self-reported youth surveys included:

1. OCHS-EBS measures for Social Phobia (SP), Generalized Anxiety Disorder (GAD), Major Depressive Disorder (MDE), Attention Deficit Hyperactivity Disorder (ADHD), Oppositional Defiant Disorder (ODD), Conduct Disorder (CD).
2. K6 for general psychological distress.
3. Psychosis symptoms score.

Conservative non-parametric Kendall's Tau correlations (τb) were performed, whereby $p<0.05$ indicated significant correlations.

Substance use was significantly related to more frequent and severe self-reported mental health symptomatology. In summary:

1. Externalizing symptoms (ODD & CD) were associated with higher frequencies of substance use across all substance types, using alcohol for coping purposes, and Alcohol Use Disorder Identification Test (AUDIT) scores.
2. Internalizing symptoms (SP, GAD, MDE, K6) were associated with prescription drug misuse and coping motives for alcohol and cannabis.

**Kendall's tau_b correlations (chart review severity)**		suicidal ideation, plan, & attempt	Non-suicidal self-harm	Aggressive threats & behaviours
Cannabis use	Correlation Coefficient	0.167	0.128	.227*
	Sig. (2-tailed)	0.057	0.159	0.016
	N	98	94	87
Alcohol use	Correlation Coefficient	0.15	0.063	.212*
	Sig. (2-tailed)	0.087	0.486	0.025
	N	98	94	87
Tobacco use	Correlation Coefficient	.227*	0.142	.324**
	Sig. (2-tailed)	0.011	0.126	0.001
	N	98	94	87
E-cigarette use	Correlation Coefficient	0.094	0.092	0.136
	Sig. (2-tailed)	0.295	0.322	0.159
	N	98	94	87
Prescription drug misuse	Correlation Coefficient	0.136	0.113	.262**
	Sig. (2-tailed)	0.142	0.24	0.009
	N	98	94	87
Illicit drug misuse	Correlation Coefficient	0.044	0.131	.282**
	Sig. (2-tailed)	0.631	0.169	0.005
	N	98	94	87

Cannabis Coping Motives Score	Correlation Coefficient	-.059	-.012	-.068
	Sig. (2-tailed)	.636	.921	.595
	N	44	44	41
Alcohol Coping Motives Score	Correlation Coefficient	-.019	.117	.159
	Sig. (2-tailed)	.881	.347	.215
	N	45	45	42
CUDIT	Correlation Coefficient	.160	-.110	.039
	Sig. (2-tailed)	.165	.347	.749
	N	49	49	44
AUDIT	Correlation Coefficient	.148	.094	.281*
	Sig. (2-tailed)	.206	.431	.022
	N	48	47	44
Using cannabis with others	Correlation Coefficient	-.073	-.126	-.085
	Sig. (2-tailed)	.574	.342	.530
	N	44	44	41
Using alcohol with others	Correlation Coefficient	-.074	-.205	-.113
	Sig. (2-tailed)	.582	.130	.416
	N	45	45	42

**Kendall's tau_b correlations (self-report severity)**		SP	GAD	MDE	ADHD	ODD	CD	Psychosis	K6
Cannabis use (n=100)	Correlation Coefficient	-0.076	0.007	0.075	0.128	.266**	.309**	0.028	0.035
	Sig. (2-tailed)	0.346	0.93	0.344	0.102	0.001	0	0.721	0.652
Alcohol use (n=100)	Correlation Coefficient	0.054	0.038	0.105	0.073	.210**	.250**	0.059	0.152
	Sig. (2-tailed)	0.502	0.632	0.186	0.35	0.008	0.002	0.456	0.051
Tobacco use (n=100)	Correlation Coefficient	0.005	0.069	0.071	.185*	.295**	.447**	0.133	0.065
	Sig. (2-tailed)	0.952	0.397	0.377	0.021	0	0	0.099	0.412
E-cigarette use a(n=100)	Correlation Coefficient	-0.021	0.05	0.107	0.14	.259**	.300**	0.083	0.115
	Sig. (2-tailed)	0.802	0.543	0.188	0.081	0.001	0	0.304	0.149
Prescription drug misuse (n=100)	Correlation Coefficient	.208*	.245**	.279**	.168*	.230**	.319**	0.116	.195*
	Sig. (2-tailed)	0.014	0.004	0.001	0.042	0.005	0	0.163	0.017
Illicit drug use (n=100)	Correlation Coefficient	0.081	0.137	.174*	.206*	.274**	.374**	0.129	0.04
	Sig. (2-tailed)	0.338	0.102	0.037	0.012	0.001	0	0.122	0.627
Cannabis coping motives (n=45)	Correlation Coefficient	.293**	.398**	0.206	0.001	0.052	0.04	-0.014	.301**
	Sig. (2-tailed)	0.009	0	0.065	0.992	0.635	0.714	0.897	0.006
Alcohol coping motives (n=46)	Correlation Coefficient	0.128	.302**	.236*	.320**	.299**	.316**	0.137	.276*
	Sig. (2-tailed)	0.259	0.007	0.033	0.004	0.007	0.004	0.216	0.012
AUDIT total score (n=49)	Correlation Coefficient	0.02	0.123	0.186	.289**	.336**	.444**	.364**	0.157
	Sig. (2-tailed)	0.853	0.25	0.077	0.006	0.001	0	0.001	0.134
CUDIT total score (n=50)	Correlation Coefficient	-0.094	0.015	0.072	-0.041	0.002	-0.005	0.136	0.105
	Sig. (2-tailed)	0.371	0.885	0.488	0.686	0.987	0.96	0.189	0.304
Using cannabis with others (n=45)	Correlation Coefficient	-0.172	-0.14	-0.112	0.049	0.054	0.108	-0.164	-0.138
	Sig. (2-tailed)	0.148	0.234	0.34	0.671	0.642	0.352	0.159	0.234
Using alcohol with others (n=46)	Correlation Coefficient	-0.103	-0.167	-0.067	-0.111	-0.143	-0.117	-.470**	-.298*
	Sig. (2-tailed)	0.405	0.174	0.579	0.353	0.232	0.325	0	0.013

Part B: Substance use and clinical severity at follow-up

6-month follow-up self-reported psychiatric symptoms as per the OCHS-EBS were predicted by frequency of the most commonly used substances at index admission (i.e., cannabis, alcohol, tobacco cigarettes, and e-cigarettes) and whether youth increased, decreased, or kept their use the same at 6-month follow-up. Linear regressions were performed adjusting for index psychiatric symptoms.

	Follow-up Internalizing Symptoms		Follow-up Externalizing Symptoms		Follow-up Total symptoms	
Baseline Cannabis	1.9 (0.7) p=0.013	2.2 (0.8) p=0.008	0.89 (0.99) p=0.372	1.1 (1.0) p=0.297	2.4 (1.5) p=0.032	3.8 (1.6) p=0.023
Increased (n=10)		0.5 (2.1) p=0.809		2.9 (2.6) p=0.256		3.9 (4.0) p=0.333
Decreased (n=8)		-3.0 (2.5) p=0.232		-2.4 (3.0) p=0.426		-5.0 (4.7) p=0.288
Baseline Alcohol	1.3 (0.7) p=0.091	1.6 (0.9) p=0.066	0.8 (0.9) p=0.373	1.5 (1.1) p=0.189	2.5 (1.4) p=0.096	3.6 (1.7) p=0.038
Increased (n=13)		0.1 (2.1) p=0.977		-0.6 (2.5) p=0.805		-0.9 (4.0) p=0.830
Decreased (n=15)		-1.9 (2.4) p=0.443		-3.0 (2.8) p=0.293		-5.8 (4.4) p=0.199
Baseline Tobacco cigarettes	2.2 (0.8) p=0.006	1.7 (0.9) p=0.066	1.9 (1.0) p=0.059	1.7 (1.2) p=0.162	4.6 (1.5) p=0.004	4.1 (1.8) p=0.031
Increased (n=7)		2.9 (2.5) p=0.24		3.4 (2.9) p=0.251		6.1 (4.6) p=0.187
Decreased (n=9)		1.9 (2.5) p=0.451		0.7 (3.1) p=0.833		2.3 (4.8) p=0.629
Baseline E-cigarettes	1.8 (0.7) p=0.018	1.9 (0.7) p=0.016	0.5 (0.9) p=0.599	0.8 (0.9) p=0.394	2.7 (1.5) =0.077	3.0 (1.4) p=0.043
Increased (n=11)		4.0 (2.0) p=0.050		5.4 (2.5) p=0.034		10.4 (3.7) p=0.008
Decreased (n=8)		0.5 (2.3) p=0.842		-0.4 (3.0) p=0.890		1.5 (4.5) p=0.734

6. Substance use and clinical complexity

Indicators of clinical psychiatric complexity were operationalized based on clinician-diagnoses from chart reviews and based on youth self-reported surveys.

Complexity indicators from the charts included:

- Number of diagnoses at discharge
- Number of categories of diagnoses at discharge (excluding other)
    - Categories included: anxiety and OCD, depressive related, cluster-B/BPD, trauma and stressor related, ADHD and neurodevelopmental, Eating Disorders, Problems with family relations, and SUDs.

Complexity indicators from the youth survey included:

- Number of OCHS-EBS symptom scores surpassing population thresholds
    - Total number (i.e., GAD, SP, MDE, ADHD, ODD, and CD)
    - # of internalizing (i.e., GAD, SP, MDE)
    - # externalizing (i.e., ADHD, ODD, CD)
    - INT & EXT

With the exception of INT&EXT, conservative non-parametric Kendall's Tau correlations (τb) were performed, whereby $p<0.05$ indicated significant correlations. Point-biserial (r_{pb}) were used for INT&EXT correlations.

**Kendall's tau & Point-biserial correlations for complexity**		self-report total count	self-report int count	self-report ext count	self-report int & ext	total physician count	category physician count
Cannabis use (n=100)	Correlation Coefficient	.212**	0.029	.289**	.209*	0.04	0.117
	Sig. (2-tailed)	0.009	0.727	0.001	0.037	0.63	0.162
Alcohol use (n=100)	Correlation Coefficient	.200*	0.09	.215*	0.181	0.009	0.093
	Sig. (2-tailed)	0.013	0.285	0.012	0.071	0.911	0.269
Tobacco use (n=100)	Correlation Coefficient	.280**	0.049	.367**	.357**	0.122	0.167
	Sig. (2-tailed)	0.001	0.571	0	0	0.147	0.05
E-cigarette use (n=100)	Correlation Coefficient	.284**	0.083	.324**	.286**	0.074	0.141
	Sig. (2-tailed)	0.001	0.335	0	0.004	0.382	0.1
Prescription drug misuse (n=100)	Correlation Coefficient	.360**	.334**	.229*	.321**	.173*	.197*
	Sig. (2-tailed)	0	0	0.012	0.001	0.047	0.026
Illicit drug use (n=100)	Correlation Coefficient	.341**	.191*	.317**	.273**	0.114	.200*
	Sig. (2-tailed)	0	0.031	0	0.006	0.189	0.023
Cannabis coping motives (n=45)	Correlation Coefficient	.229*	.383**	-0.02	.331*	0.091	.358*
	Sig. (2-tailed)	0.044	0.001	0.868	0.027	0.43	0.016
Alcohol Coping Motives (n=46)	Correlation Coefficient	.412**	0.222	.372**	.539**	.297*	.540**
	Sig. (2-tailed)	0	0.058	0.002	0	0.01	0
CUDIT total score (n=50)	Correlation Coefficient	0.005	0.07	-0.029	0.126	0.12	0.116
	Sig. (2-tailed)	0.959	0.52	0.791	0.382	0.268	0.422
AUDIT total score (n=49)	Correlation Coefficient	.396**	0.132	.431**	.417**	0.121	.442**
	Sig. (2-tailed)	0	0.237	0	0.003	0.271	0.001
Using cannabis with others (n=45)	Correlation Coefficient	-0.043	-0.137	0.103	0.054	-0.064	0.049
	Sig. (2-tailed)	0.721	0.27	0.41	0.723	0.598	0.747
Using alcohol with others (n=46)	Correlation Coefficient	-0.187	-0.138	-0.134	-0.224	-0.12	-0.205
	Sig. (2-tailed)	0.129	0.28	0.298	0.135	0.34	0.172

7. Substance use and service use

Methods

Repeat ED visits and psychiatric hospitalizations were obtained through hospital administrative records. Timelines for analyses were operationalized as: (1) 3 years prior to index; (2) 6 months prior to index; and (3) 6 months after index. ED visits and psychiatric hospitalizations were analyzed separately. Any mental health related ED visit was created through combining mental health and substance use ED visits with psychiatric ED visits. Any mental health inpatient admissions were identified by combining discharges from the Child and Youth Mental Health Inpatient unit and the Eating Disorder Inpatient unit. There may have been some youth who were discharged from other non-mental health related units who were missed – i.e., transferred to a medical unit prior to discharge – however, this is extremely rare and typically it occurs in the reverse whereby youth are medically cleared on a medical unit first and subsequently transferred to the mental health unit.

Logistic regressions were conducted predicting any ED visit or any inpatient admission. All analyses were adjusted for whether the index admission was a direct admission (proxy for out of city) or through the local ED. Independent variables included: substance use frequencies (measure categorically and continuously), cannabis and alcohol coping motives scores, CUDIT and AUDIT scores, and proportion of time spent using cannabis and alcohol with others (whereby lower scores are reflective of a greater frequency of solitary use).

Results

When looking at substance use frequencies, all substances (i.e., alcohol, cannabis, tobacco, e-cigarettes, prescription drugs, and illicit drugs) were significantly related to a higher likelihood of having had an inpatient admission in the 3 years prior. Prescription drug misuse was additionally related to a higher likelihood of ED presentations in the 3 years prior. E-cigarette use was additionally related to a higher likelihood of an ED visit 6 months after index. For cannabis and alcohol, coping motives and AUDIT/CUDIT scores were not related to hospital contacts. However, using alcohol alone was related to a greater likelihood of re-presenting to ED within 6 months and a having a prior inpatient admission in the past 3 years and 6 months. Using cannabis alone was related to a greater likelihood of an ED visit in the past 3 years and 6 months. Other visit types and timelines were not significant. Please see the table for full results.

Logistic regressions predicting any ED visit or any inpatient admission. All analyses adjusted for whether the index admission was a direct admission or through local ED. Presented as adjusted OR (95% CI); p value.

	**ED visits**			**Inpatient Admissions**		
	**3-year prior**	**6-months prior**	**6-months post**	**3-year prior**	**6-months prior**	**6-months post**
Substance use Factors						
**Cannabis**						
not past month (ref=never)	2.66 (0.81,8.77); p=0.108	1.83 (0.53,6.29); p=0.338	3.33 (0.91,12.12); p=0.069	7.65 (2.15,27.22); p=0.002	4.03 (1.01,16.08); p=0.048	1.71 (0.52,5.62); p=0.38
past month but not every day (ref=never)	1.43 (0.47,4.32); p=0.53	0.52 (0.13,2.06); p=0.353	1.66 (0.45,6.07); p=0.444	4.19 (1.31,13.41); p=0.016	2.57 (0.66,10.02); p=0.175	0.46 (0.13,1.71); p=0.249
daily (ref=never)	1.95 (0.57,6.68); p=0.289	0.8 (0.19,3.32); p=0.758	1.18 (0.27,5.18); p=0.828	3.65 (1.01,13.25); p=0.048	0.87 (0.14,5.34); p=0.883	0.54 (0.13,2.32); p=0.407
Continuous frequency score	1.18 (0.81,1.73); p=0.392	0.83 (0.54,1.29); p=0.412	1.03 (0.67,1.58); p=0.891	1.45 (1,2.11); p=0.048	1.03 (0.66,1.59); p=0.899	0.75 (0.49,1.15); p=0.191
Cannabis coping motives	0.99 (0.9,1.08); p=0.763	1 (0.9,1.12); p=0.965	1.08 (0.97,1.21); p=0.178	1.1 (0.99,1.21); p=0.062	1.01 (0.91,1.13); p=0.81	1.11 (0.95,1.28); p=0.186
CUDIT score	1.03 (0.95,1.12); p=0.47	1.02 (0.93,1.13); p=0.664	0.95 (0.87,1.04); p=0.274	1.06 (0.98,1.14); p=0.174	0.98 (0.89,1.07); p=0.615	1.03 (0.93,1.15); p=0.566
Frequency of use with others	0.58 (0.35,0.96); p=0.035	0.51 (0.26,1); p=0.049	0.62 (0.35,1.08); p=0.088	0.7 (0.44,1.11); p=0.126	0.56 (0.3,1.02); p=0.058	0.74 (0.4,1.37); p=0.336
**Alcohol**						
not past month (ref=never)	4.49 (1.3,15.47); p=0.017	1.29 (0.34,4.82); p=0.709	3.8 (0.98,14.71); p=0.053	10.87 (2.86,41.3); p=0	2.4 (0.53,10.91); p=0.258	1.26 (0.39,3.99); p=0.701
past month but not heavy (ref=never)	2.17 (0.56,8.34); p=0.261	1.41 (0.33,6.05); p=0.646	0.86 (0.16,4.53); p=0.86	3.14 (0.73,13.47); p=0.123	2.42 (0.46,12.62); p=0.294	0.73 (0.18,2.93); p=0.652
past month heavy (ref=never)	1.36 (0.42,4.41); p=0.612	0.87 (0.24,3.2); p=0.832	1.32 (0.35,5.04); p=0.684	4.69 (1.28,17.21); p=0.02	2.8 (0.65,12.08); p=0.168	0.27 (0.06,1.16); p=0.078
Continuous frequency score	1 (0.7,1.44); p=0.983	0.95 (0.64,1.43); p=0.813	0.94 (0.63,1.41); p=0.775	1.31 (0.92,1.85); p=0.135	1.31 (0.86,2.01); p=0.207	0.67 (0.44,1.01); p=0.057
Alcohol coping motives	0.98 (0.9,1.06); p=0.631	0.94 (0.85,1.03); p=0.172	1.06 (0.97,1.16); p=0.207	1.1 (1.01,1.2); p=0.023	1.06 (0.97,1.16); p=0.204	1.12 (0.99,1.27); p=0.085
AUDIT score	0.95 (0.85,1.07); p=0.397	0.95 (0.83,1.07); p=0.374	1.04 (0.93,1.16); p=0.486	1.06 (0.97,1.17); p=0.206	1.08 (0.97,1.2); p=0.15	0.98 (0.87,1.11); p=0.775

Frequency of use with others	0.79 (0.46,1.36); p=0.399	0.61 (0.35,1.07); p=0.083	0.52 (0.29,0.92); p=0.025	0.52 (0.29,0.92); p=0.025	0.42 (0.22,0.78); p=0.006	0.53 (0.27,1.04); p=0.066
**Tobacco cigarettes**						
less than monthly (ref=never)	1.08 (0.36,3.21); p=0.89	1.66 (0.51,5.35); p=0.398	1.89 (0.57,6.25); p=0.296	1.85 (0.65,5.31); p=0.25	1.54 (0.44,5.35); p=0.499	1.67 (0.55,5.07); p=0.369
monthly but less than daily (ref=never)	0.93 (0.24,3.59); p=0.921	0.96 (0.21,4.38); p=0.961	2.57 (0.62,10.64); p=0.193	3.45 (0.94,12.6); p=0.061	1.58 (0.35,7.12); p=0.552	1.11 (0.26,4.78); p=0.887
daily (ref=never)	2.15 (0.6,7.72); p=0.24	0.52 (0.1,2.76); p=0.439	0.99 (0.22,4.47); p=0.99	4.46 (1.28,15.54); p=0.019	2.01 (0.5,8); p=0.322	0.56 (0.11,2.83); p=0.479
frequency	1.2 (0.82,1.77); p=0.348	0.88 (0.56,1.37); p=0.564	1.14 (0.74,1.74); p=0.553	1.7 (1.16,2.49); p=0.007	1.26 (0.82,1.93); p=0.289	0.91 (0.59,1.4); p=0.654
**E-cigarettes**						
less than monthly (ref=never)	4.63 (1.04,20.62); p=0.045	2.18 (0.55,8.58); p=0.267	2.06 (0.47,8.95); p=0.337	3.11 (0.87,11.13); p=0.081	1.42 (0.32,6.21); p=0.642	0.63 (0.12,3.22); p=0.577
monthly but less than daily (ref=never)	0.6 (0.17,2.11); p=0.426	0.67 (0.16,2.83); p=0.584	1.99 (0.52,7.64); p=0.317	1.94 (0.61,6.17); p=0.262	1.54 (0.41,5.81); p=0.528	1.14 (0.31,4.16); p=0.84
daily (ref=never)	0.99 (0.27,3.63); p=0.987	0.63 (0.12,3.35); p=0.584	5.76 (1.31,25.4); p=0.021	3.04 (0.9,10.2); p=0.072	1.35 (0.31,5.82); p=0.692	1.26 (0.34,4.72); p=0.732
frequency	0.95 (0.65,1.38); p=0.783	0.87 (0.55,1.37); p=0.549	1.67 (1.08,2.58); p=0.022	1.44 (1.01,2.07); p=0.046	1.14 (0.75,1.75); p=0.535	1.07 (0.72,1.6); p=0.737
**Prescription drug misuse**						
not past 3 months (ref=never)	5.86 (1.28,26.87); p=0.023	1.23 (0.27,5.71); p=0.79	1.24 (0.26,5.79); p=0.785	2.63 (0.72,9.64); p=0.145	0.99 (0.19,5.23); p=0.988	0.57 (0.11,2.9); p=0.497
past 3 months (ref=never)	1.28 (0.46,3.52); p=0.635	0.74 (0.24,2.3); p=0.598	1.13 (0.38,3.32); p=0.832	3.16 (1.18,8.49); p=0.022	1.6 (0.53,4.82); p=0.401	0.51 (0.15,1.71); p=0.272
**Illicit and other drug use**						
not past 3 months (ref=never)	2.53 (0.78,8.2); p=0.121	1.5 (0.42,5.31); p=0.53	1.68 (0.47,6.07); p=0.429	7.41 (2.14,25.68); p=0.002	1.97 (0.57,6.79); p=0.282	1.52 (0.46,5.04); p=0.497
past 3 months (ref=never)	0.93 (0.3,2.95); p=0.905	0.89 (0.24,3.3); p=0.862	2.47 (0.74,8.3); p=0.143	1.57 (0.52,4.72); p=0.423	1.34 (0.37,4.88); p=0.662	1.52 (0.46,5.04); p=0.495

8. Staff survey detailed summary of results

There was an 86% response rate (37/43) with almost half RNs (49%) and half CYWs (51%). Differences between RNs and CYWs are to be expected in all domains given different scopes of practice, however both RNs and CYWs have a high degree of patient exposure and thus increased knowledge and confidence related to substance use would be prudent across both roles. Over half (54%) of the staff participating in the survey had been working on the unit 5 years or longer with only 2 staff reporting less than 1 year on the unit. Given the mandate of this unit is outside the scope of a substance treatment facility, specialized substance use training is not a requirement for the staff. As such, 84% of the staff had never received specialized substance use training. All closed ended questions had no missing data (with the exception of one item missing one respondent). Open ended questions were answered by 63% to 83% of the sample. Open-ended responses ranged from 1 to 6 sentences or bullet points, with typical responses to all questions being 2-3 sentences.

A majority of staff reported feeling fairly or completely **confident** in their knowledge of how alcohol, cannabis, and nicotine/tobacco impacted youth on the unit (RNs 56 to 78%; CYWs 68 to 74%), however, for other prescription and illicit drug misuse a minority of staff reported high confidence (RNs 22 to 50%; CYWs 16 to 37%). Open ended responses further elucidated higher confidence in assessing and addressing alcohol, cannabis, and tobacco/nicotine may be due to more frequent use by youth and existing protocols for Nicotine Replacement Therapy (NRT) on the unit and a greater need for education on other drugs and co-use of substances. Overall, staff expressed perceived importance of considering both occasional and regular substance use in the clinical conceptualization of youth admitted to the unit.

All staff indicated at least one barrier to a hypothetical incorporation of comprehensive substance use **assessments**, with the most common barriers being lack of training (RNs 72%; CYWs 95%) and time pressures (RNs 72%; CYWs 63%). No staff stated they felt uncomfortable talking about substances. Of note, RNs indicated conducting substance use screening during admission but identified areas to improve comprehensiveness including more question prompts and space to document in the electronic medical record (EMR). Thus, many staff indicated that adding designated space in the Kardex (i.e., patient summary sheet) and EMRs alongside training of assessments and interventions for those who screen positive would facilitate comprehensive assessments if deemed necessary and appropriate for an inpatient setting.

Regarding **withdrawal**, roughly half of RNs reported feeling confident in identifying and responding to withdrawal for alcohol, cannabis, nicotine, opioids, and sedatives (50 to 67%) while less than one third (28%) reported confidence for other illicit drugs. CYWs were explicitly asked about psychotherapeutic and supportive withdrawal interventions, and few reported high levels of confidence across all substances (11 to 39%). RNs reported existing withdrawal protocols, including COWS (opioids), CIWA (alcohol and benzos), and NRT (nicotine). Both

RNs and CYWs reported uncertainty regarding non-pharmacological management of withdrawal.

Very few staff reported high levels of confidence in delivering brief psychoeducation or brief motivational **interventions**. Of RNs, 50% reported high levels of confidence for psychoeducation related to tobacco/nicotine (in alignment with existing protocols) while for the other substances, high confidence in psychoeducation was only reported by 17-33%. High confidence in psychoeducation was also highest for tobacco/nicotine among CYWS (47%) while only 21% and 26% reported high confidence for psychoeducation related to alcohol and cannabis respectively and no CYWs reported high confidence related to opioids, sedatives, or other drugs. Related to brief motivational interventions, no staff reported being completely confident with only 22% of RNs and 16% of CYWs reporting feeling fairly confident. Staff do lead a bi-weekly group on substance use which uses evidence-based strategies focused predominantly on alcohol, cannabis, and nicotine. Staff made suggestions for more training and standardization regarding facilitating appropriate conversations about substance use in the milieu, more psychoeducational materials, and more targeted interventions for youth using substances.

Overall: (1) staff believe substance use is important and common among youth on the unit and want to improve how they assess and address substance use (e.g., withdrawal management, brief interventions, unit structures and programming); (2) staff have ideas about how to facilitate improvements in quality of care including greater standardization of assessments and interventions, separate cohorting and staffing for youth with more severe co-occurring problems, more direct substance related interventions, and more indirect facilitation of appropriate and supportive conversations; and (3) staff are open to and want more education and training to increase knowledge, confidence, and standardization of practices. Specific quotes supporting these domains available upon request.

Percentage of staff endorsing each barrier			
**Barriers**	**RNs**	**CYWs**	**All staff**
**Lack of training**	**72%**	**95%**	**81%**
**Time pressures**	**72%**	**63%**	**68%**
Unfamiliar with treatment resources in the community	56%	53%	54%
Youth do not often tell the truth about their substance use	33%	58%	43%
Do not know what to do if youth screen positive while on the unit	39%	37%	38%
Screening for substance use is the function of other health services	11%	37%	24%
Uncertainty regarding the effectiveness of available treatments	11%	26%	19%
Lack of funds to make system changes	6%	32%	19%
Do not want youth to worry about who will be informed about their substance use	11%	16%	14%
Lack of space and privacy for conversations	6%	11%	8%
Documentation of substance use problems in the medical record may adversely affect youth	0%	11%	5%
Personally uncomfortable talking about substance use with youth	0%	0%	0%
I do not foresee any barriers to changing screening procedures	0%	0%	0%

Percentage of staff endorsing each facilitator			
**Facilitators**	**RNs**	**CYWs**	**All staff**
Adding a space in the Kardex to flag substance use concerns	**89%**	**89%**	**89%**
Training on how to deliver psychoeducation	**83%**	**95%**	**89%**
Training on psychotherapeutic approaches for addressing substance use	**83%**	**89%**	**87%**
Adding specific questions to the electronic medical record	**72%**	**89%**	**84%**
Training on pharmacological options for addressing substance use	**89%**	**79%**	**84%**
Training on how to ask questions related to substance use	**72%**	**79%**	**76%**

Confidence Reported by Registered Nurses

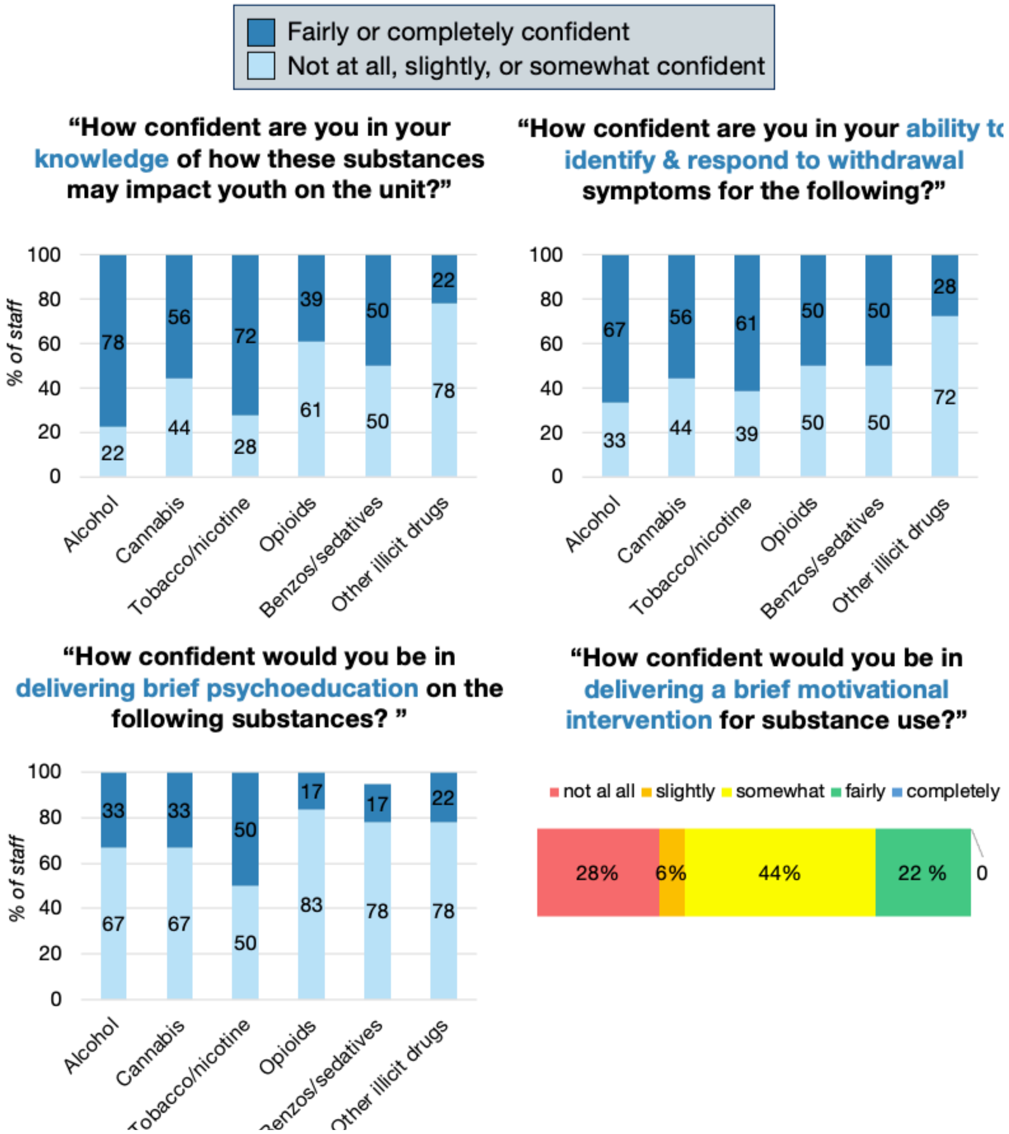

Confidence Reported by Child and Youth Workers

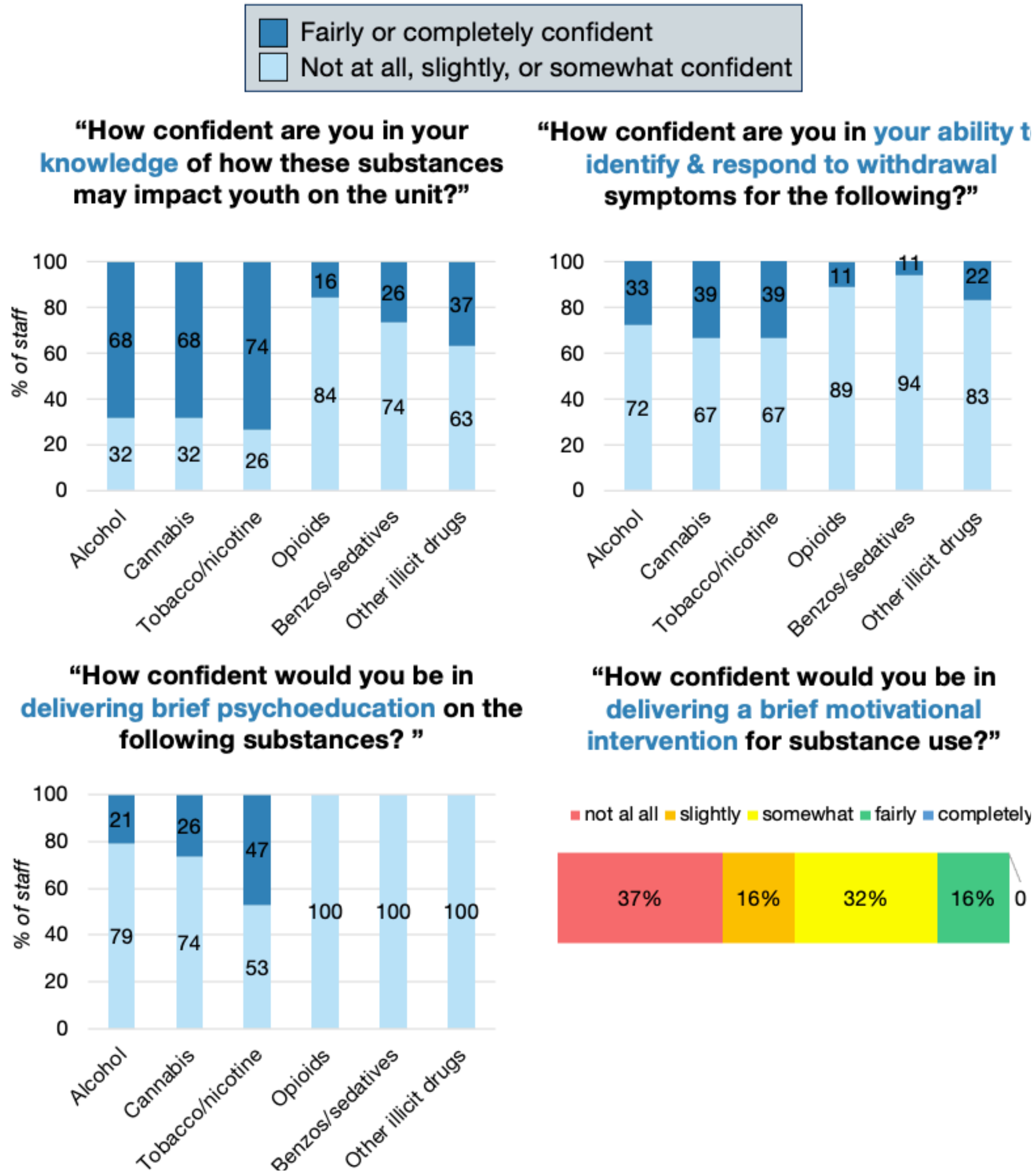

Supplement: Supplementary file 5 — Additional file 5. Detailed methods and results. [file 13034_2021_403_MOESM5_ESM.pdf]
